# Spatially resolved, high-dimensional transcriptomics sorts out the evolution of biphasic malignant pleural mesothelioma: new paradigms for immunotherapy

**DOI:** 10.1186/s12943-023-01816-9

**Published:** 2023-07-17

**Authors:** F Torricelli, B Donati, F Reggiani, V Manicardi, S Piana, R Valli, F Lococo, Alessia Ciarrocchi

**Affiliations:** 1 Laboratory of Translational Research, Azienda Unità Sanitaria Locale-IRCCS di Reggio Emilia, Reggio Emilia, 42123 Italy; 2 Pathology Unit, Azienda Unità Sanitaria Locale-IRCCS di Reggio Emilia, Reggio Emilia, 42123 Italy; 3 Thoracic Surgery Unit, IRCCS-Fondazione Policlinico Gemelli, Roma, Italia; 4 Catholic University of the Sacred Heart, Roma, Italia

**Keywords:** Malignant pleural mesothelioma, Cancer heterogeneity, Tumor microenvironment, Inflammation, Epithelial mesenchymal transition, Tumor associated Macrophages

## Abstract

**Background:**

Malignant Pleural Mesothelioma (MPM) is a dreadful disease escaping the classical genetic model of cancer evolution and characterized by wide heterogeneity and transcriptional plasticity. Clinical evolution of MPM is marked by a progressive transdifferentiation that converts well differentiated epithelioid (E) cells into undifferentiated and pleomorphic sarcomatoid (S) phenotypes. Catching the way this transition takes place is necessary to understand how MPM develops and progresses and it is mandatory to improve patients’ management and life expectancy. Bulk transcriptomic approaches, while providing a significant overview, failed to resolve the timing of this evolution and to identify the hierarchy of molecular events through which this transition takes place.

**Methods:**

We applied a spatially resolved, high-dimensional transcriptomic approach to study MPM morphological evolution. 139 regions across 8 biphasic MPMs (B-MPMs) were profiled using the GeoMx™Digital Spatial Profiler to reconstruct the positional context of transcriptional activities and the spatial topology of MPM cells interactions. Validation was conducted on an independent large cohort of 84 MPMs by targeted digital barcoding analysis.

**Results:**

Our results demonstrated the existence of a complex circular ecosystem in which, within a strong asbestos-driven inflammatory environment, MPM and immune cells affect each other to support S-transdifferentiation. We also showed that TGFB1 polarized M2-Tumor Associated Macrophages foster immune evasion and that TGFB1 expression correlates with reduced survival probability.

**Conclusions:**

Besides providing crucial insights into the multidimensional interactions governing MPM clinical evolution, these results open new perspectives to improve the use of immunotherapy in this disease.

**Supplementary Information:**

The online version contains supplementary material available at 10.1186/s12943-023-01816-9.

## Introduction

Malignant pleural mesothelioma (MPM) is a rare and incurable cancer, which incidence is increasing in many countries [1]. MPM escapes the classical genetic model of cancer evolution, lacking a distinctive genetic fingerprint [2]. Omics profiling revealed extensive tumor heterogeneity [3–7], failing to identify major vulnerabilities and restraining the development of MPM-oriented therapies.

Morphologically, the MPM heterogeneity is evidenced by the existence of three distinct phenotypes: epithelioid (E), sarcomatoid (S) and biphasic MPM (B) which consists of a mix of epithelioid and sarcomatoid components [8, 9]. B-MPM is itself a highly heterogeneous entity in which the overall extension of the S-component affects features and clinical behavior of the lesion [1, 10].

Indeed, the degree of MPM cell differentiation reflects aggressiveness with sarcomatoid lesions being the most lethal form. For this reason, despite many limitations including lack of standardization and sampling biases, histology remains the most credited prognostic criteria and a tool for treatment choice [10].

However, MPMs remain difficult to manage, making urgent the development of more sophisticated and accurate tools that catching the real degree of heterogeneity of the lesions can overcome these limitations and provide a more reliable and effective prognosis scoring system [6, 8].

E-MPM and S-MPM do not represent separated entities but the two extreme conditions of a cell transdifferentiation process that converts well-differentiated MPM cells into scarcely differentiated phenotypes promoting aggressiveness. B-MPMs represent this transition “while is taking place”, constituting the formal proof of this process and the link between the two extreme histological conditions.

Deep transcriptional profiling also supports this hypothesis indicating the presence of E-like and S-like populations in each MPM and picturing MPM lesions as a continuous gradient of phenotypes in between the E- and S- condition [3].

Besides, unsupervised clustering analysis, based on transcriptional profiling revealed that over 60% of histologically classified E-MPMs are transcriptionally identified as biphasic entities, suggesting that the transitional state is indeed much more diffuse than expected based on standard histological evaluation [5].

Epithelial-to-mesenchymal transition (EMT), the process through which epithelial cells shed their structural organization to acquire mesenchymal features including motility and resistance to stress stimuli [2, 11], has been recognized as main driver of the E to S MPM transdifferentiation [12, 13]. However, what ignites this transition and how this transition takes place remain far from being elucidated.

Transition is a matter of timing, but catching the timing in cancer is a quite complicated matter. The use of bulk transcriptomic approaches, while providing a useful measurement of the overall extent of the transition does not allow to resolve timing nor to catch the consequentiality of the molecular events during this process. Until then, we lack fundamental clues to correctly picture MPM heterogeneity and to fully understand this disease.

Here, we used for the first time a spatial transcriptomic approach in a retrospective series of B-MPMs to follow the E to S transition and to reconstruct the molecular events that trigger and support this process.

Besides, providing new insights into the mechanisms driving this process, we evidenced a functional association between a pro-inflammatory immune-environment and the transcriptional rewiring that drives and supports this phenotypical transdifferentation.

## Materials and methods

### Patients cohorts and study design

A retrospective cohort of consecutive MPM patients was retrieved from the Pathology Unit of our Institution between 2010 and 2019. Inclusion criteria were age > 18 years, availability of Formalin Fixed Paraffin Embedded (FFPE) tumor tissues and follow up information. Histological sections of all samples were revised by two different pathologists. 8 B-MPM from surgical resection were employed for the spatial transcriptomic analysis (Traning Set). 84 MPMs were used for the validation analysis (Validation Set), all 84 yielded RNA of quality and quantity sufficient for the gene expression analysis. Clinical data, pathological features, and long-term follow-up information were systematically reviewed and recorded in Table 1.

**Table 1:** Clinical information of MPMs included in both training and validation cohorts

	Training set	Validation set	
	Overall (N = 8)	Overall (N = 84)	
**Sex**			
F	2 (25.0%)	17 (20.2%)	
M	6 (75.0%)	67 (79.8%)	
**Age**			
Mean (SD)	73.5 (8.8)	70.6 (8.6)	
**Side**			
Left	2 (25.0%)	29 (35.8%)	
Right	6 (75.0%)	52 (64.2%)	
N-Miss		3	
**Surgery**			
Biopsy	5 (62.5%)	49 (59.0%)	
Pleurectomy	3 (37.5%)	34 (41.0%)	
N-Miss		1	
**Histology**			
Epithelioid	0 (0%)	34 (40.5%)	
Biphasic	8 (100.0%)	32 (38.1%)	
Sarcomatoid	0 (0%)	18 (21.4%)	
**Stage**			
I	4 (50%)	38 (47.6%)	
II	0 (0%)	4 (5.0%)	
III	0 (0%)	7 (8.7%)	
IV	4 (50%)	31 (38.8%)	
N-Miss		4	
**Death**			
No	2 (25%)	8 (9.6%)	
Yes	6 (75%)	75 (90.4%)	
N-Miss		1	
**Overall survival (months)**			
Mean (SD)	11.9 (8.1)	20.3 (20.6)	
N-Miss		3	

### Spatial transcriptomics

Spatial Transcriptomics was performed by GeoMx DSP® (Nanostring technologies, USA) [14] starting from slides of 5 μm FFPE tumor tissue. One slide for each sample was analyzed and a mean of 23 circular area of interest (AOIs) (range 15–32) was collected for each sample. The slides were hybridized with the GeoMx® Cancer Transcriptome Atlas panel according to manufacturer protocol. This panel includes RNA probes for 1834 genes for comprehensive profiling of tumor biology and tumor microenvironment, covering canonical cancer pathways, immune cell types and checkpoint molecules. In order to perform spatial selection, slides were stained with GeoMx Solid Tumor TME Morphology Kit including antibodies against Cytokeratins (Pan-CK) and nuclear stain SYTO-13.

For each slide AOIs were selected by two pathologists (SP, RV) as representative of the tumor heterogeneity, based on morphology and expression of the Pan-CK marker. Three types of regions were selected: pure E-AOIs corresponding to area including only epithelioid cells, pure S-AOIs (pS-AOIs) corresponding to area including only sarcomatoid cells and transitional regions containing both mixed epithelioid and sarcomatoids cells. These transitional regions were further segmented to separate the E (tE-AOIs) and S component (tS-AOIs). Hybridized RNA-probes were collected from each of the selected AOIs, and libraries for NGS sequencing were constructed by GeoMx® Seq Code Pack (Nanostring technologies). Libraries were pooled according to AOIs dimension, purified by AMPure XP beads (Beckman Coulter, Brea, California, USA) clean up and finally resuspended in a volume proportional to the number of pooled AOIs. The quality and quantity of library pools were evaluated by Agilent Bioanalyzer. Libraries were diluted to 1.6pM and sequenced by Illumina NextSeq500 (paired-end 2 × 27), expecting for each library/AOI a coverage of at least 30 reads for µm^2^ of collected region. After demultiplexing, FastQ files were converted into DCC files by GeoMx® Next-generation sequencing (NGS) Pipeline App available in BaseSpace Illumina Sequence Hub and then uploaded on the GeoMx DSP platform to be associated with the corresponding AOIs.

### GeoMx DSP data analysis

First, quality control tests (QC) were performed on AOIs to evaluate sequencing, number of nuclei collected and background effect. The thresholds used for AOIs selection were as follow: raw reads = 1000, aligned reads = 80, sequencing saturation parameter = 40%, negative probe count mean = 4.5, minimum nuclei count = 100, no template control count = 100. AOIs displaying value below these thresholds in any of the selecting criteria were excluded. Then, QC on probes was conducted according to the following parameters: ratio across all segment = 0.1, percentage AOIs threshold (Grubbs test) = 20, standard deviation amount for the LOQ = 2. Successively, segments that expressed less than 20% of genes and genes expressed in less than 10% of the AOIs were filtered out and raw gene counts were normalized on geometric mean of all target genes.

### Differential analysis

All spatial transcriptomics analyses were performed on GeoMx DSP platform. After completion of the normalization process, we compared the expression profiles of E-AOIs and S-AOIs or pE-AOIs vs. pS-AOIs and tE-AOIs vs. tS-AOIs. For each comparison, significantly deregulated genes were selected by considering an adjusted p-value < 0.05 and an absolute log2 of the Fold Change (log2FC) > 0.1.

### Gene ontology

Gene ontology (GO) enrichment analysis was performed on Biological Process subontology by EnrichR online software. Protein-protein interactions were represented by STRING (v11.5) online software. Significance threshold was set at adjusted p-value < 0.05 (Benjamini-Hochberg correction).

### Cellular deconvolution

Cellular deconvolution was conducted on the basis of normalized gene expression profiles applying “SpatialDecon” pipeline, downloaded from GeoScript Hub (https://nanostring.com/products/geomx-digital-spatial-profiler/geoscript-hub/), on GeoMx DSP platform. Scaled abundance score of each cell type was compared between E-AOIs and S-AOIs and p-value was calculated by two-tailed Student’s t test.

### Data validation by nanostring nCounter

nCounter analysis was performed as previously described [15, 16]. Total RNA was extracted using Maxwell® RSC RNA FFPE kit (Promega) from five 5 μm FFPE slides. RNA was assessed by NanoDrop 2000 (Thermo Fisher Scientific). 27 MPM samples yielded RNAs that didn’t reach the required standard for the analysis (A260/A280 ≥ 1.7 and A260/A230 ≥ 1.8) and were excluded. In the remaining 84 samples the expression of a specifically designed custom panel including 217 target and 15 housekeeping genes was evaluated by nCounter (NanoString Technologies) following manufacturers’ protocol. Data from nCounter were analyzed by nSolver Analysis Software 4.0 (NanoString Technologies). After imaging quality control, raw gene counts were normalized on technical controls and three housekeeping genes as previously described [15, 16]. Fold changes were calculated as the ratio between B-MPMs and E-MPMs and between S-MPMs and E-MPMs, p-value was calculated by two-tailed Student’s t test and adjusted by Benjamini–Hochberg method.

### Statistical analysis

Bioinformatic and statistical analyses on gene expression profiles were conducted by R Software v4.2.2. Single gene expression comparisons between the four AOIs groups were performed by Wilcoxon test. Correlation analyses were performed by Spearman test. All correlation plots, boxplots and histograms were performed using “ggplot2” (v3.4.2) and “ggpubr” R packages(v0.4.0). R packages “fmsb” (v0.7.5) and “circlize” (v0.4.15) were used for radarplot and chordplot representation,respectively.

Heatmaps were produced using “Pheatmap” R package (v1.0.12).

For survival analyses patients were dichotomized on the basis of first and fourth quartile of TGFB1 expression and p-value was calculated by Log-rank test. Survival plot was created using “Survival” (v3.2-11) and “Survminer” R (v 0.4.9) packages and multivariate analysis was performed applying Cox Proportional-Hazards model.

Tests were considered statistically significant with a p-values < 0.05. Adjusted p-values were calculated applying “Benjamini-Hochberg” method.

## Results

### Gene expression spatial resolution recapitulates the timing of transition identifying early and late stages

**Fig. 1:**
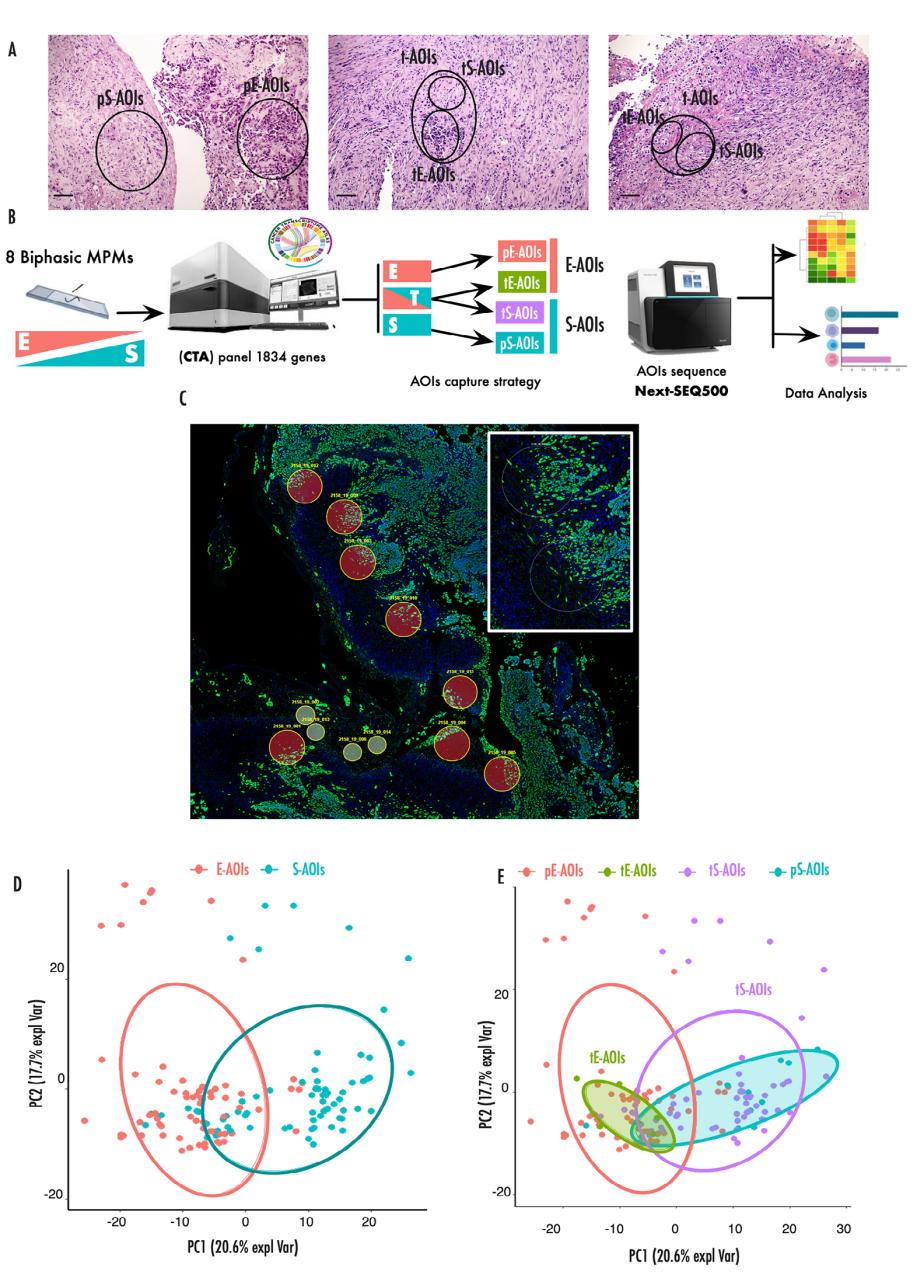
Morphology guided spatial transcriptomics on B-MPM tissues **(A)** Representative H&E staining of B-MPMs included in the training set. Magnification 100X. **(B)** Experimental design of the spatial transcriptomic analysis. Three types of regions were selected based on cell morphology and expression of Pan-CK marker. pE-AOIs, pS-AOIs corresponded to area of pure epithelioid and sarcomatoid component respectively. Transitional regions (t-AOIs) containing both mixed epithelioid and sarcomatoid cells were further segmented in tE-AOIs and tS-AOIs. Collected AOIs were sequenced and transcriptional profiles analysed. **(C)** GeoMx DSP scan showing AOIs from a representative B-MPM collected from a surgical biopsy. Large circles indicate segmented transitional AOIs while small circles circle indicate pure AOIs. Insight displays the enlargement of representative transitional areas. **(D)** Principal component analysis (PCA) distribution of total E-AOIs and S-AOIs, based on differentially expressed genes (adjusted p-value < 0.05). **(E)** Principal component analysis distribution of the indicated AOIs, based on differentially expressed genes (adjusted p-value < 0.05)

Study design is summarized in Fig. 1A-B. Three types of area were selected: pure E-AOIs (pE-AOIs) corresponding to area including only epithelioid cells, pure S-AOIs (pS-AOI) corresponding to area including only sarcomatoid cells and transitional AOIs (t-AOIs) containing both mixed epithelioid and sarcomatoid cells. Overall 8 pE-AOIs, 11 pS-AOIs, and 60 t-AOIs were selected starting from 8 surgically resected B-MPMs. The t-AOIs were further segmented to separate the E (tE-AOIs, N = 60) and S component (tS-AOIs, N = 60) resulting in a total of 120 AOIs.

Transcriptional profiling for each of these regions was performed (Fig. 1C and Supplementary Fig. 1A). PCA analysis showed that gene expression profile efficiently segregated the overall E- and S-AOIs confirming the accuracy of the spatial selection (Fig. 1D).

We assumed that pE-AOIs represented the initial stage and pS-AOIs the final stage, while t-AOIs were intermediate stages. PCA (Fig. 1E) and correlation analysis (Supplementary Fig. 1B) of the gene expression profile of the 4 groups showed high heterogeneity. As expected pE-AOIs and pS-AOIs tended to segregate at the widest distance (Supplementary Fig. 1C), confirming the profound transcriptional differences between these two groups. tE-AOIs aggregated in close proximity with pE-AOIs while tS-AOIs displayed the highest degree of heterogeneity among the 4 groups spreading from E to S. Collectively these data indicate that tumor cells localized at the transition borders represent early stages of the transition and precursors of the fully transdifferentiated sarcomatoid cells.

### Transcriptional rewiring underlines extracellular matrix (ECM) remodeling and supports massive structural reorganization during the E to S transition

**Fig. 2:**
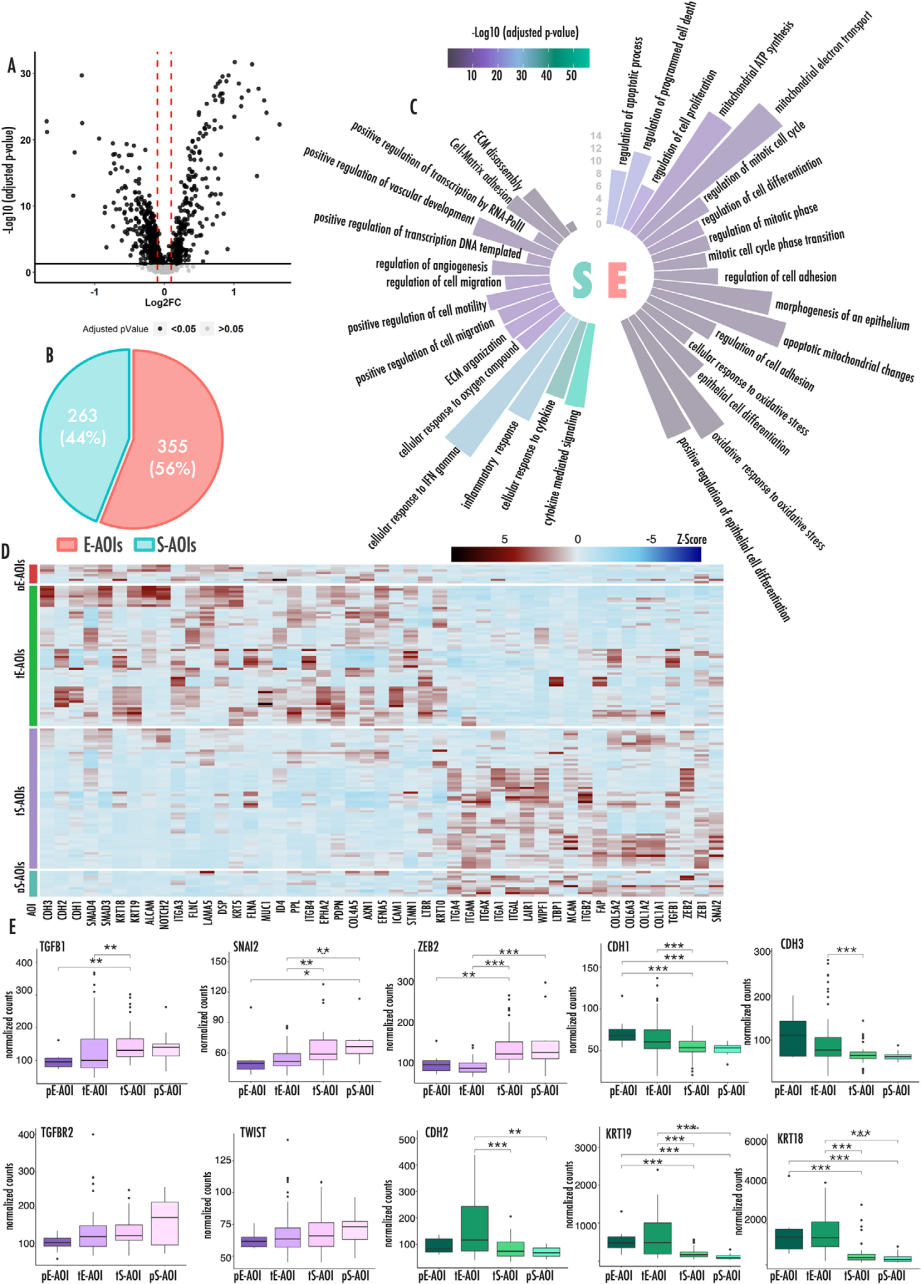
Principal biological processes involved in E to S transition **(A)** Volcano plot showing differentially expressed genes between E-AOIs and S-AOIs. Black dots represent significantly deregulated genes (adjusted p-value < 0.05). Red lines represent fold change threshold (FC) |0.1| **(B)** Pie chart showing the number and relative percentage of differentially expressed genes upregulated in E-AOIs and S-AOIs. **(C)** Circular histograms representing principal biological processes upregulated in Epithelioid (right) and Sarcomatoid (left) regions of interests. Color legend expresses the level of significance of each enriched category. The graduated axis represents the fraction of genes involved in each biological process and deregulated in our setting. **(D)** Heatmap representing expression of all deregulated EMT-TFs and markers in the 4 AOI groups. Color gradient expresses the z-score of each gene in each sample. **(E)** Box plots representing the expression level of EMT markers (green) and upstream regulators (pink) across the different AOIs. In this figure p-values are represented as follow: *<0.05, **<0.01 ***<0.001

We performed a differential analysis between the overall E- and S-AOIs. 618 genes resulted significantly deregulated (Fig. 2A) (Supplementary Table 1). Of these 355 were upregulated in E-AOIs as compared to S-AOIs while 263 genes were more expressed in the S-AOIs as compared to the E counterpart (Fig. 2B). We performed a GO analysis to connect these genes to specific biological processes (Fig. 2C). We observed that E-AOIs genes were enriched in processes involved in epithelial differentiation, cell-cell junction, cell death and mitosis. We also observed a relevant node of genes involved in mitochondrial homeostasis and oxidative cell metabolism indicating a potential metabolic rewiring (Fig. 2C and Supplementary Fig. 2A). By contrast, S-AOIs genes were enriched in extracellular matrix organization and interaction, cell motility, locomotion and angiogenesis (Fig. 2C and Supplementary Fig. 2B). Of note, transcription and gene expression regulation were among the top scoring categories in this analysis with several transcription factors and chromatin regulators upregulated in S-AOIs. Noticeably, SNAI2, ZEB1, ZEB2 and TWIST master transcriptional drivers of EMT were all found upregulated in S-AOIs (Supplementary Tables 1, Supplementary Fig. 2C-D). A similar analysis was performed to compare pE-AOIs vs. pS-AOIs and tE-AOIs vs. tS-AOIs (Supplementary Fig. 3 and Supplementary Tables 3–6).

The overall picture emerging by this analysis evoked a massive structural reorganization of MPM cells during the E to S transition supported by a deep rewiring of the gene expression program, primed by the activation of EMT-associated transcription factors (TFs). Indeed, graphical representation of EMT genes expression recapitulates the different AOIs along the entire transdifferentiation process (Fig. 2D).

We also explored the expression trend of a representative set of EMT-associated genes in the four groups of AOIs focusing on both upstream regulators (pink) and phenotypical markers of this process (green) (Fig. 2E).

We observed that the expression of epithelial phenotypical markers decreased significantly moving from tE-AOIs to tS-AOIs and further to pS-AOIs, but not between pE and tE-AOIs. By contrast, upstream regulators tend to increase consistently across all passages, confirming that their activation anticipate the structural changes associated with EMT and evidenced at the level of morphology. Differently from what expected, CDH2 known to be a mesenchymal marker during EMT, followed the expression of CDH1 and CDH3 epithelial markers, decreasing during the transition instead of being associated with the S-phenotype.

### E to S transition is associated with inflammation and increased immune infiltration

**Fig. 3:**
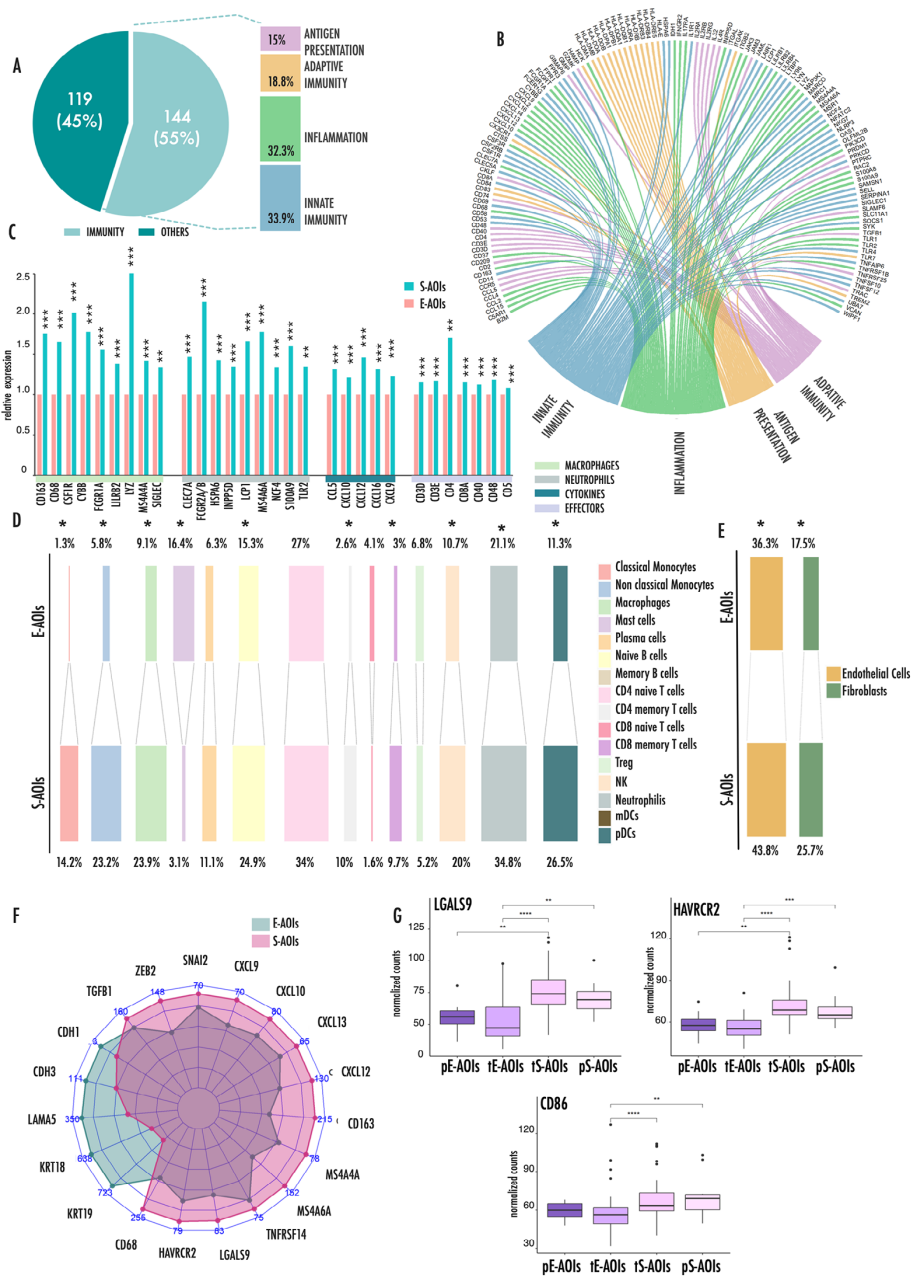
Inflammation primes immune cell infiltration during E-to S transition **(A)** Graphical representation the distribution of genes upregulated in S-AOIs as compared to E- AOIs in our analysis. Pie Chart displays the percentage of immune-related genes in this list on the total. Histogram represents the percentage distribution of immune related genes in the four main immune related processes. Some of the genes were included in more than one category, according to their multiple functions. **(B)** Chord plot represents the connections between immune related genes upregulated in S-AOIs and the four main immune related processes. **(C)** Histograms representing the relative expression of selected markers of immune populations. Data are expressed as fold change between the average expression in the S-AOIs as compared to the average expression in the E-AOIs. **(D)** Deconvolution results, obtained from gene expression data, showing scaled abundance percentage of each immune cell population in E- and S-AOIs. **(E)** Deconvolution results, obtained from gene expression data, showing scaled abundance percentage of fibroblasts and endothelial cells in E- and S-AOIs. **(F)** Radar plot showing the average expression level of principal pro-inflammatory and EMT genes in S- and E-AOIs. **(G)** Box plots representing the expression levels of LGALS9, HAVRCR2 and CD86 in the four types of AOIs during E to S transition. In this figure p-values are represented as follow: *<0.05, **<0.01 ***<0.001

We noticed that a significant proportion (55%) of genes upregulated in the S-AOIs were involved in inflammation and immune cells modulation (Fig. 3A and Supplementary Fig. 4). Specifically, these genes were engaged in innate and adaptive immunity (33.9% and 18.8%, respectively), inflammation (32.3%) or antigen presentation (15%) (Fig. 3A-B). In particular, several cytokines, chemokines and interleukins and their associated receptors were highly represented in the list of genes upregulated in S-AOIs (Fig. 3B, Supplementary Table 7).

During inflammation these molecules are known to be produced by tumor and stroma cells but also by granulocytes [17]. Indeed, macrophages and neutrophils specific genes governing granulocytes activation, including de-granulation and release of pro-inflammatory signals, were found in the list of genes upregulated in S-AOIs (Fig. 3C).

All these elements are known to create a hot microenvironment that primes the recruitment and activation of other immune effector cells. Surface markers expressed by lymphocytes, including both CD4+/CD8 + T-cells and B-Cells, were found to be associated with the S component.

To support these observations, we applied a deconvolution analysis to our dataset to reconstruct, based on gene expression, the functional map of the immune cells during the E to S transition (Fig. 3D). This analysis confirmed a deep reorganization of the immune landscape during transition. Granulocytes populations including macrophages, neutrophils and monocytes were all significantly more represented in the S-AOIs as compared to the E-AOIs. Accordingly, main effector immune cells including CD4 + and CD8 + T cells, B cells and Natural Killers (NKs) were also more represented in the S-compartment than in the E counterpart. In line with the enrichment of genes involved in the antigen presentation, plasmacytoid dendritic cells (pDCs) were enriched in the S-AOIs as compared with E-AOIs. By contrast, T regulatory cells (T-regs) and mast cells were more frequently detected within the E-AOIs. We also observed a significant increase in the percentage of fibroblasts and endothelial cells in the S-AOIs (Fig. 3E) which is coherent with the strong ECM remodeling and boosted angiogenetic process that we observed (Fig. 2C). Radar plot in Fig. 3F summarizes these data showing how the pro-inflammatory signature overrepresented in the S-compartment segregates with the expression of EMT drivers but it appeared complementary to the expression of phenotypical epithelial markers.

In light of these data, we investigated the activation state of immune cells in the S compartment looking for potential immune evasion signals (Fig. 3G and Supplementary Fig. 5). Noticeably, several immune checkpoints were significantly overexpressed in the S-AOIs as compared to E-AOIs, such as the T-cell-specific inhibitory receptor HAVCR2 and its corresponding tumor ligand LGALS9, or the inhibitory CD86 that interacts with CTLA4 on T-cells (Fig. 3G). Distribution of their expression across the four phenotypical stages, showed that all these molecules ramped moving from tE- to tS-AOIs at the early stages of progression within the transitional regions. Their expression remained high at the end of the process in the pS-AOIs. These observations indicated that the widespread immune infiltrate associated with the S-component likely resides in a dysfunctional state of exhaustion, driven by the chronic persistence of tumor antigens and the immune tolerance-related mechanisms induced by cancer cells.

### Tumor associated macrophages (TAM) EMT and immune evasion in B-MPM

**Fig. 4:**
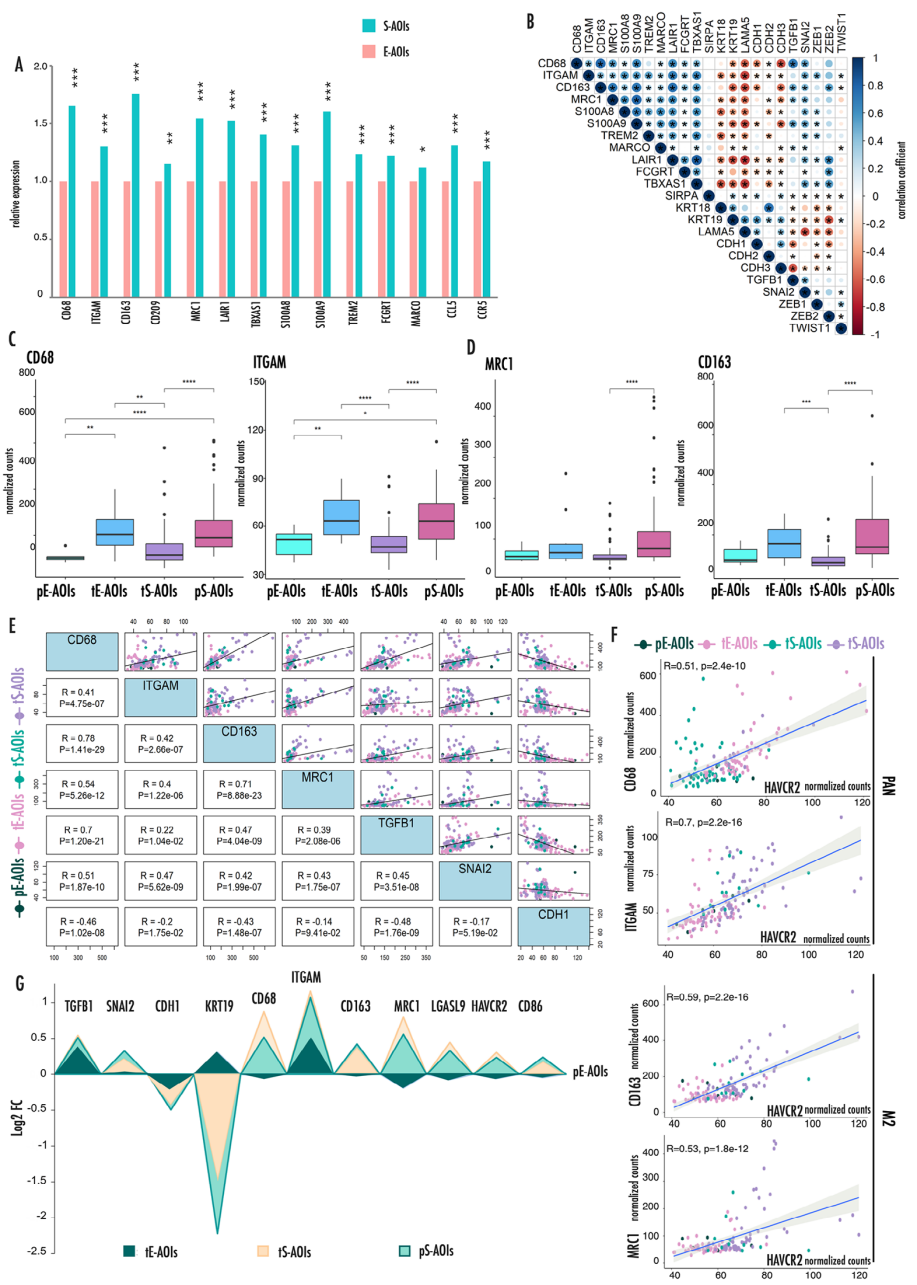
M2 type TAMs are associated to EMT and expression of T-cells associated immune checkpoints **(A)** Histograms representing the relative expression of TAM associated markers. Data are expressed as fold change between the average expression in the S-AOIs as compared to the average expression in the E-AOIs. **(B)** Correlation matrix showing expression correlations between EMT- markers and TAM-associated genes. Asterisks mark significantly associated correlations. The color intensity and the size of the circles are proportional to the correlation coefficients. (**C-D)** Box plots representing the expression level of pan-macrophages markers **(C)** and M2 selective markers **(D)** in the four types of AOIs during E to S transition. **(E)** Correlation distribution across all AOIs between EMT drivers (TGF1B1, SNAI2) and the epithelial marker CDH1 with both pan-macrophages and M2 selective markers. For each gene comparison, the correlation plot is represented on the right and the correlation coefficient with relative p-value is reported on the left. Axes values refer to normalized counts. **(F)** Scatter plots showing the direct correlation of pan-macrophages and M2 selective markers with HAVCR2 expression across all AOIs. G) Area chart summarizing the relative expression of EMT-associated genes, TAM markers and immune checkpoints in tE-, tS- and pS- AOIs as compared to pE-AOIs

Tumor associated macrophages (TAM) are known to contribute substantially to inflammation and immune modulation [18–20]. Mounting evidence is reporting a supportive role of these cells in driving aggressiveness and metastatic spreading of several cancers including MPM, by influencing tissue architecture and promoting among the others cell motility, matrix reorganization, epithelial and mesenchymal cell phenotype and positioning. Coherently, in our analysis, several TAM associated-genes were robustly upregulated in S-AOIs as compared with E-AOIs confirming a potential contribution of these cells to the E to S transition (Fig. 4A).

Correlation matrix highlights how TAM genes were strongly associated with EMT drivers and inversely correlated with epithelial markers confirming the positive link between these cells and the EMT process that occurs during MPM progression (Fig. 4B).

Following the distribution of these genes in the four types of AOIs, we observed that the expression of CD68 and ITGAM (CD11b) considered pan-macrophages markers increases at the very early stages of transition and continues to grow throughout the entire process (Fig. 4C). By contrast M2 selective markers, CD163 and MRC1 (CD206) expression increases later during this process coherently with a progressive polarization of the macrophage population toward the M2 phenotype (M2-TAM) [21] during transition (Fig. 4D).

TGFB1 is known to partake in TAM homeostasis and to promote their polarization toward the pro-tumorigenic M2 phenotype [22]. To investigate this potential link in our setting, we explored the specific association of TGFB1 and TAM markers in our dataset (Fig. 4E, Supplementary Fig. 6). We observed a striking positive correlation between TAM genes and TGFB1. Also, TAM markers showed a positive correlation with SNAI2 downstream effector of the TGFB1 signal but a negative correlation with the expression of the epithelial marker CDH1. The highest correlation was observed between TGFB1 and CD68 (R = 0.7, p = 2.2e-16). Also, CD163 (R = 0.47, p = 4.04e-09) and MRC1 (R = 0.39, p = 2.08e-06) showed a positive correlation with TGFB1, coherently with the role of TGFB1 in driving the M2-TAM polarization.

M2-TAM are known to partake in cancer immune evasion strategies by modulating the expression of T-cell specific checkpoints. Noticeably, we observed a strong positive correlation between M2-TAM genes and the expression of the immune checkpoint gene HAVCR2 (Fig. 4F). Similar results were obtained for LGASL9 and CD86, remarking a potentially crucial role of these cells in silencing the immune system during cancer progression (Supplementary Fig. 7). Figure 4G summarizes these data, showing the trend of the reported genes in the 3 AOIs types indicated relatively to their level of expression on pE-AOIs.

### Validation analysis in a separate cohort of MPM

**Fig. 5:**
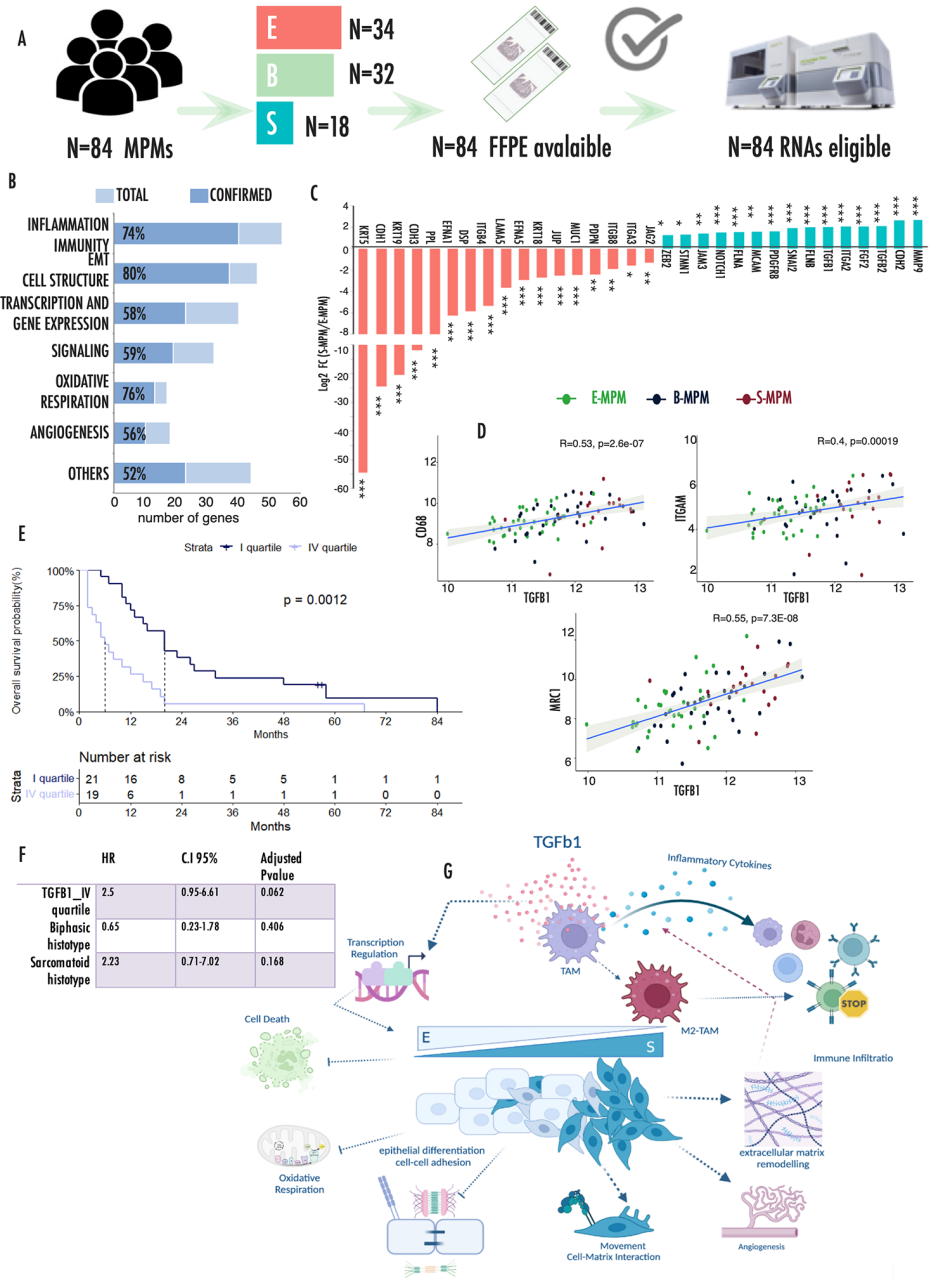
Validation analysis in an independent cohort of MPMs **(A)** Pipeline of samples analysis by nCounter Nanostring technology. **(B)** Bar chart representing the number of deregulated genes involved in each functional category. Percentages represent the fraction of validated genes in each category. X-axes report the total number of genes included in the panel for each category as reported in Supplementary Table 8. **(C)** Histograms representing the relative expression of EMT associated genes in S-MPM samples as compared to E-MPMs. **(D)** Correlation plots showing the direct correlation of CD68, ITGAM and MRC1 with TGFB1 expression. Axes values refer to normalized counts. **(E)** Kaplan Meier curves representing the significantly different overall survival of patients with low (I quartile) and high (IV quartile) expression of TGFB1. In this figure p-values are represented as follow: *<0.05, **<0.01 ***<0.001. **(F)** Multivariate Cox analysis including TGFB1 expression quartile and histotype in the validation cohort. **(G)** Schematic representation model of the results emerged from the analysis

We confirmed these data in an independent cohort of MPMs by analyzing the expression of a target panel of 217 genes using a digital analytic procedure (Fig. 5A). 84 samples yielded RNA of quality and quantity compatible with the analysis of which 34 E-MPMs, 32 B-MPM and 18 S-MPM. Clinical features of this cohort are summarized in Table 1. Genes included in this analysis were selected among those differentially expressed in the spatial transcriptional profiling, in order to represent the most relevant pathways emerged from our analysis including EMT and cell structure, inflammation and immunity, transcription regulation, angiogenesis and oxidative cell metabolism (Supplementary Table 2). Figure 5B shows the overall rate of genes per each category that were confirmed in this analysis. This analysis confirmed that during transition, loss of epithelial features in S-MPMs was associated with gain of mesenchymal markers and in particular with the over expression of TGFB1 and EMT-drivers TFs (including ZEB2 and SNAI2) (Fig. 5C). Genes associated with EMT showed an intermediate expression in B-MPMs as compared to E- and S-MPMs (Supplementary Fig. 8A), coherently with their transitional state, and displayed a trend of association with the percentage of sarcomatoid tissue in the samples (Supplementary Fig. 8B). Of note, CDH2, FLNA and FLNB mesenchymal markers observed upregulated in the E-AOIs in spatial transcriptomic analysis of B-MPMs were in this analysis more expressed in the S-MPM lesions. We also validated the observation that S-MPMs are associated with increased inflammation and immune infiltration as indicated by the increased expression of immune cell markers including CD4 and CD8 and TAM-associated genes in S-MPMs (Supplementary Fig. 5). Noticeably, the greatest increase was observed for MRC1, strengthening the hypothesis of a relevant polarization of M2-TAM during the E to S transition. Coherently with the spatial transcriptomic profiling, correlation analysis between TGFB1 and TAM markers across all samples confirmed a significant positive association (Fig. 5D). TGFB1 was significantly correlated with both pan and M2-specific TAM markers, strengthening the functional link between this growth factor and this immune cell population and further highlighting the relevance of this balance during MPM progression. Taken together our data, point to TGFB1 as pivotal player during MPM progression. Kaplan Meier curve (Fig. 5E) showed that TGFB1 expression significantly correlated with reduced survival probability in the entire cohort of MPMs, confirming the central role of this growth factor in the definition of MPM clinical progression and aggressiveness. Noticeably, TGFB1 remained associated with worse prognosis even in a multivariate analysis including histotypes, supporting the role of this factor in dictating MPM aggressiveness (Fig. 5F).

These results confirm that TGFB1 contributes to this process playing a dual function: modelling cell architecture by inducing EMT and shaping MPM immune microenvironment by influencing TAM polarization and their immune suppressive activity (Fig. 5G).

## Discussion

MPM is a heterogeneous disease, characterized by a high morphological plasticity [3–5, 7]. Resolving this heterogeneity is crucial to understand what drives clinical aggressiveness and to develop new and more effective targeting strategies for MPM patients. Indeed, management of B-MPMs remains controversial, with different guidelines recommending distinct approaches. One of the main causes of this uncertainty lies in the difficulty of performing a correct evaluation of the tumor histotype at diagnosis. It is estimated that over 50% of B-MPMs are erroneously classified as E-MPMs or S-MPMs by pleural biopsy [10, 23, 24], leading to biases in the clinical choices for the management of these patients [25]. Multiple biopsies allowing a wider representation of the heterogeneity of the disease may help a more accurate evaluation of the histological phenotype. As well, improving the current knowledge about the mechanisms governing this heterogeneity may help find stratification biomarkers to support diagnosis.

Omics profiling dramatically improved the resolution of MPM heterogeneity showing that this disease cannot be categorized into precise entities but should be considered as a continuum between the two extreme morphological phenotypes. MPM, as other tumors, is a complex community in which the tissue topography and cell-cell communications govern the intimate interplay between cancer cells and their own microenvironment. Bulk transcriptomics or single cells approaches, by destroying tissue integrity, miss this level of information and produce a partial representation of the regulatory networks that control the progression of this disease [26].

In this work we used for the first time a morphology-guided spatial transcriptomic approach on a selected series of B-MPMs with the aim of resolving this transition at the transcriptional level and gaining information on how this transition takes place. This approach, by garnering gene expression information in a spatial context, allowed us to provide additional pieces of information on how MPM progression occurs [14].

Our data rendered the picture of a complex circular ecosystem in which MPM and immune cells affect each other altering the local microenvironment to support progression (Fig. 5G).

We showed that a strong inflammatory environment characterizes the appearance of the S morphology from the earliest stages of the cellular transdifferentiation.

An increased expression of inflammatory cytokines and high recruitment of inflammatory cells were observed to be associated with the S components already in the transitional regions. These signals foster the activation of both innate and adaptive immunity driving the homing to the tumor sites of numerous effector cells and in particular CD8 + T-cells. However, the chronic stimulation by tumor antigens and the persistence of the inflammatory stimuli drive these S-associated immune cells to exhaustion, as suggested by the increased expression levels of immune checkpoints including the T-cell receptor HAVCR2 and its specific ligand LGASL9.

On the other side, MPM cells undergo a profound transcriptionally-driven structural reorganization. Primed by the expression of EMT associated TFs, E-MPM cells lose their epithelial features modifying deeply their ability to interact with the surrounding environment. Cell-to-cell adhesion structures are lost and replaced by matrix interacting protein and remodeling factors.

ECM is a relevant part of the cancer ecosystem, and its dynamic changes are important during the pathogenesis and progression of this disease [27]. Stiffness and adhesive cues of ECM provide cancer cells with mechanical signals that are transduced within the cells primarily by cytoskeleton anchored proteins and affect gene expression by supporting programs that serve cells to respond to such stimuli, by altering cell architecture, fostering motility and overcoming stresses induced by the mechanical pressure [28]. Aberrant ECM remodeling is initiated by different types of stress stimuli, including oxidative stress [29].Deposition of asbestos fibers to the lungs causes the chronic release of reactive species and the production of free radicals. These highly dynamics elements may cause massive molecular instability and concur to reshape ECM during MPM progression [30]. Besides, these unstable molecules are known to mediate genotoxic signals leading to cell death. Supporting the central role of these processes during MPM evolution, our analysis indicates that the ability to handle stress induced to oxygen reactive species is central in the transition from E to S phenotype. Also, the evidence that genes upregulated in E-AOIs are enriched in mitochondrial related pathways and oxidative respiration (Supplementary Fig. 9), seems to indicate a potential metabolic switch during transition that, coherently with other setting, may serve to MPM to sustain energetic and stress adaptation [31].

Recent data reported a relevant immune-modulatory function of ECM in cancer, via the creation of niches that control the migration, localization, phenotype and function of tumor-infiltrating immune cells, ultimately contributing to the escape of immune surveillance [32]. Besides, in a recent pan cancer study, ECM-associated transcriptional program has been shown to correlate with TGFB signaling and is potentially linked to immune evasion and or adaptation [33].

Our data are in line with these observations and indicate that these dynamics are central during the morphological evolution of B-MPM and impact on clinical behavior of this disease. Using gene expression data from our internal cohort of MPMs, we demonstrated that TGFB1 is a marker of clinical aggressiveness in MPM, being associated with reduced survival probability. This can have important implications for patients’ management. Indeed, it has been observed that TGFB1 levels in pleural effusion are higher in S-MPMs as compared with E-MPMs and have strong prognostic values [34].

Our data, also identified TAMs as primary targets of TGFB1 in MPM. Macrophages are capable of displaying different and even opposing phenotypes depending on the context [22]. In cancer, TAMs in particular the M2 subtype are linked to cancer promoting function [18]. TGFB1 is known as driver of M2 polarization on TAMs in many settings and our results indicates that this also occurs in MPM. Besides inflammation and tissue remodeling, M2-TAMs have been reported to create an immune suppressive microenvironment triggering inhibitory immune checkpoints in T-cells [35–37].Coherently, our analysis highlights a predominance of M2-TAMs within immune microenvironment of B-MPM and a significant correlation between M2-TAM markers and T-cell specific immune checkpoints. Several recent works, by using widely different approaches, are raising the attention about the role of M2-TAM in MPMs [38, 39]. Ollila et al. using multiplexed fluorescence immunohistochemistry, observed that M2-TAMs are independently associated with shorter survival in a retrospective cohort of E-MPMs [40]. Creaney et al. using a comprehensive genomic immune profiling showed that MPM immune environment contained high levels of M2-TAMs associated with metalloproteinases (MMP2 and MMP14) and TGFB1 expression [41]. Lievense et al. showed that macrophages from pleural effusion of MPM patients hamper antitumor T cell immune response [42] while pharmacologically depletion of M2-TAM (with a CSF-1R Kinase Inhibitor) enhances the effectiveness of dendritic cells vaccination therapy priming antitumor immunity [43]. Our data in line with these reports further emphasizes the role of this population in MPM and pose the attention on the potential of targeting macrophages in this setting.

We are aware that our study has major limitations, first the fact that is a descriptive transcriptomic study limited to the investigation of gene transcripts. However, to the best of our knowledge this is the first report that applies a spatial transcriptomic approach to the study of B-MPM and that fitting coherently within the current literature produces a model that places many scattered pieces of evidence within a consistent framework developing new insights into the bases of this disease. The strength of this model is certified by our validation analysis that confirmed in an independent and large cohort of MPMs the obtained data, using an independent analytical procedure. Finally, even if still exploratory, these data may hold relevant clinical implications, not limited to the indication of TGFB1 as a putative prognostic marker. Recently, the introduction of immune checkpoint inhibitors (ICIs) marked a sensible improvement in the management of MPMs leading to advances in terms of survival [44–47].Of note, response to ICIs was reported to be higher in S-MPM than in E-MPM. Our data seems to provide new clues in explaining these observations laying the basis for possible strategies to optimize the employment of these drugs in this setting.

In the framework of this very aggressive and therapy orphan disease, the introduction of immune checkpoint inhibitors (ICIs) promises to mark a potential improvement in the management of MPMs leading to advances in terms of survival [47], in particular for patients with S-MPMs [48]. The reason of this discrepancy between E-MPMs and S-MPMs remains unknown and at the center of an intense debate [49–51]. While it is likely that the increased rate of response observed in S-MPM patients is determined, at least in part, by their complete refractoriness to standard chemotherapy, this cannot fully account for the sensibility to ICIs that these lesions displayed. Unraveling this knot is fundamental to maximize the effectiveness of these drugs in a setting like MPMs in which the availability of target therapies is so poor. Still, the overall impact on patients’ survival and quality of life remains arguable and highly debated, also in light of the high costs and side effects associated with these therapies [52–56]. Resolving this controversy and improving the effectiveness of these therapies for MPM patients requires a multidisciplinary and integrated effort. On one side, the implementation of rigorous methodology for the assessment of clinical benefits of these therapies in MPM patients. On the other side, a better definition of the molecular mechanisms underlining their action to provide clues to improve their use in patients.

In this framework our work offers new relevant perspectives:

1. it defines, for the first time, the functional relevance of the immune system and of the inflammatory context in the mechanisms that drive MPM evolution and identifies new details on the communication circuits between tumor and immune cells and on their topography within the lesion, therefore providing new potential candidates to exploit as predictive biomarkers of ICIs response.
2. it indicates M2-TAM polarization as an important event in the establishment of immune evasion signals, defining new potential targets.
3. it defines that these signals are not driven by “canonical” immune checkpoints.

The latest point seems of particular relevance since it offers a potential explanation to the observation that expression of PD1 and PDL1 do not predict response to ICIs in MPM. Additional studies specifically are necessary to define strategies that will improve the use of these drugs in MPMs. Our work defines for the first time a reference frame for the design of these studies.

## Electronic supplementary material

Below is the link to the electronic supplementary material.

Supplementary Material 1

## Data Availability

Data are available at Array Express E-MTAB-13043.

## Abbreviations

MPM: Malignant Pleural Mesothelioma
E-MPM: Epithelioid Malignant Pleural Mesothelioma
S-MPM: Sarcomatoid Malignant Pleural Mesothelioma
B-MPM: Biphasic Malignant Pleural Mesothelioma
EMT: Epithelial To Mesenchymal Transition
DSP: Digital Spatial Profiler
AOI: Area of Interest
CK: Citokeratin
FDR: False Discovery Rate
TGFB1: Transforming Growth Factor Beta 1
ECM: Extracellular Matrix
PCA: Principal Component Analysis
GO: Gene Ontology
TAM: Tumor Associated Macrophage
SNAI2: Snail Family Transcriptional Repressor 2
ZEB1: Zinc Finger E-Box Binding Homeobox 1
ZEB2: Zinc Finger E-Box Binding Homeobox 2
TWIST: Twist Family BHLH Transcription Factor
CDH1: Cadherin 1
CDH2: Cadherin 2
CDH3: Cadherin 3
HAVCR2: Hepatitis A Virus Cellular Receptor 2
LGALS9: Galectin 9
CTLA4: Cytotoxic T-Lymphocyte Antigen 4
ITGAM: Integrin Subunit Alpha M
MRC1: Mannose Receptor C-Type 1)
FLNA: Filamin A
FLNB: Filamin B
