# Supplementary material for: Spatially resolved, high-dimensional transcriptomics sorts out the evolution of biphasic malignant pleural mesothelioma: new paradigms for immunotherapy
Source: Mol Cancer. 2023 Jul 17;22:114. doi: 10.1186/s12943-023-01816-9 (PMC10351128; doi:10.1186/s12943-023-01816-9)
Supplement: Supplementary file 1 — Supplementary Material 1 [file 12943_2023_1816_MOESM1_ESM.pdf]

Supplementary FIGURE1

A

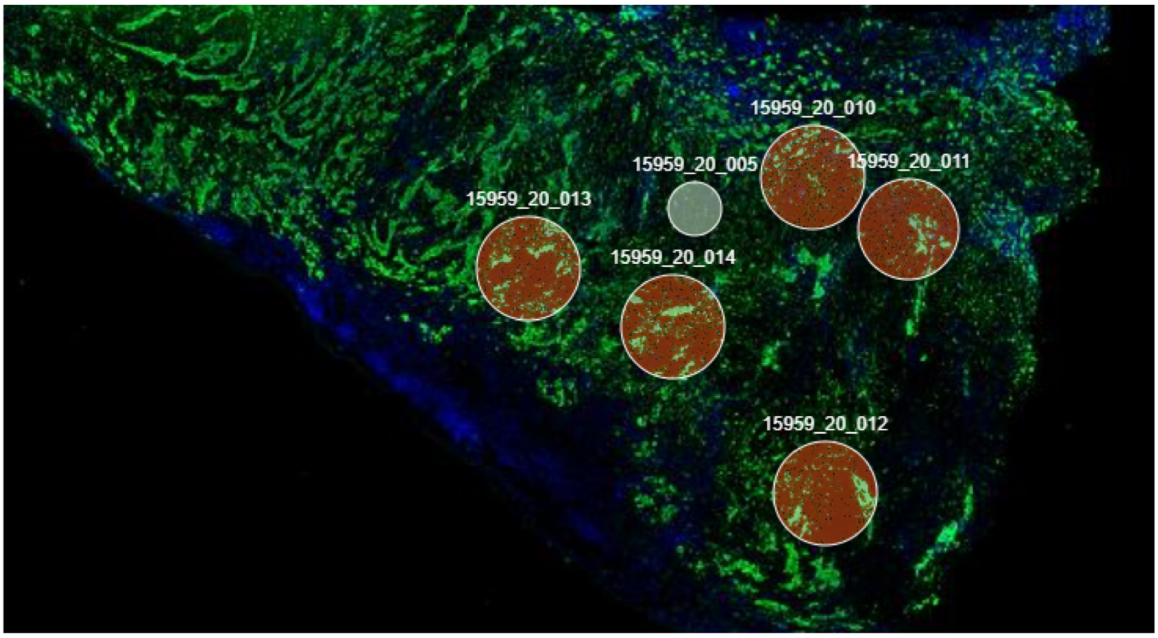

B

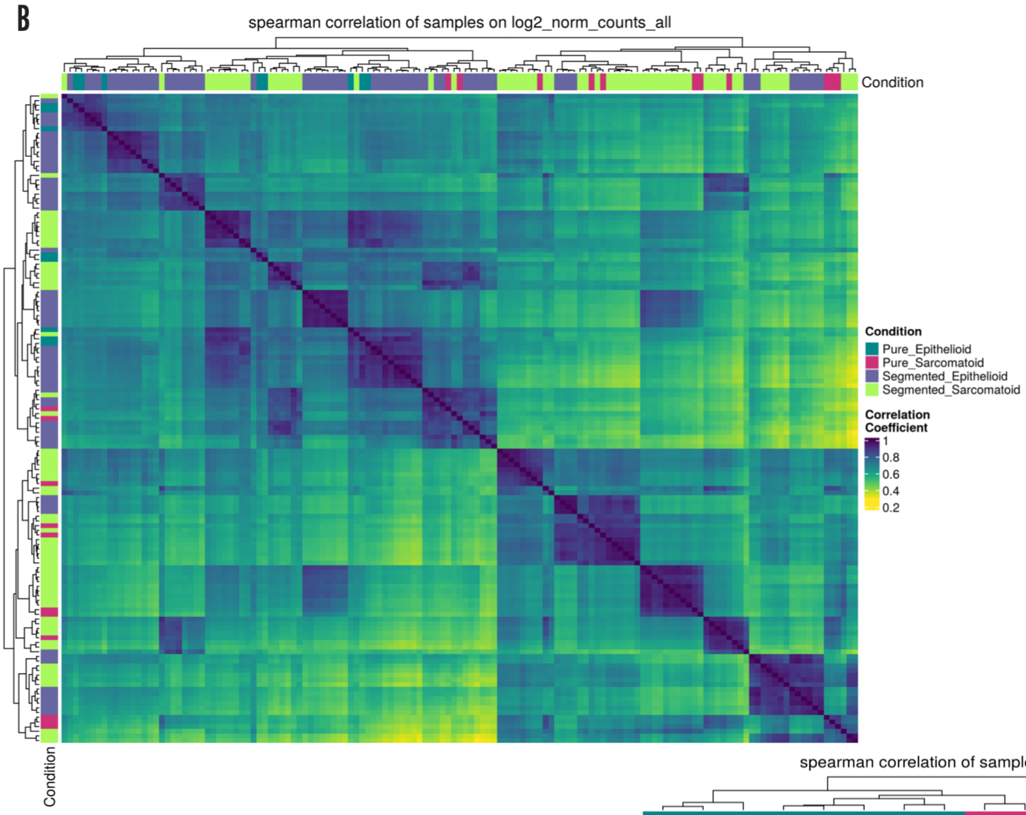

C

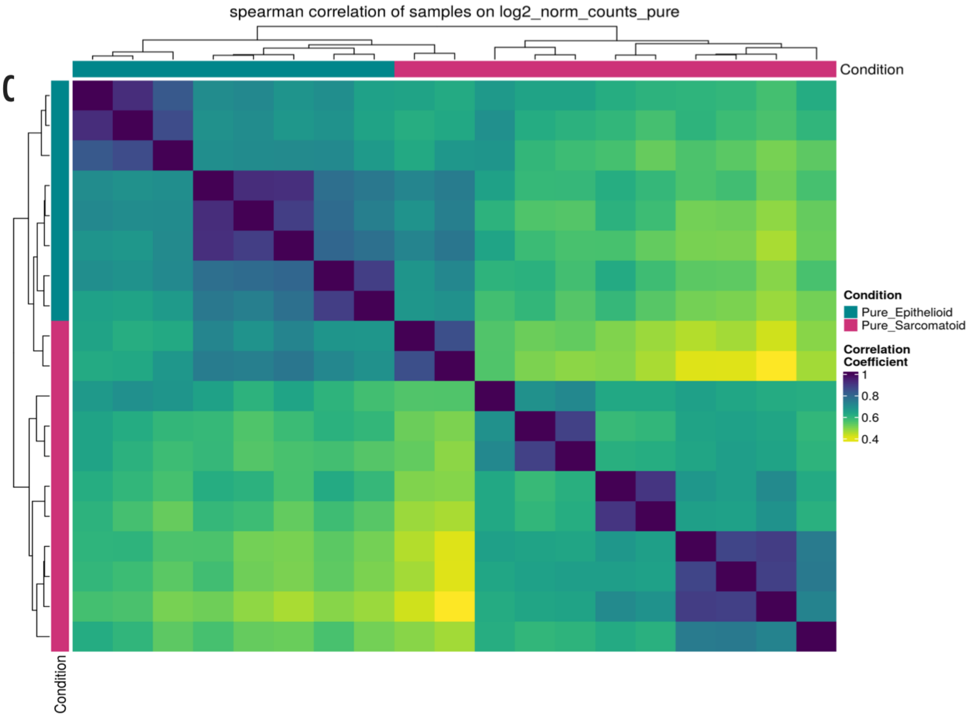

SUPPLEMENTARY FIGURE 2

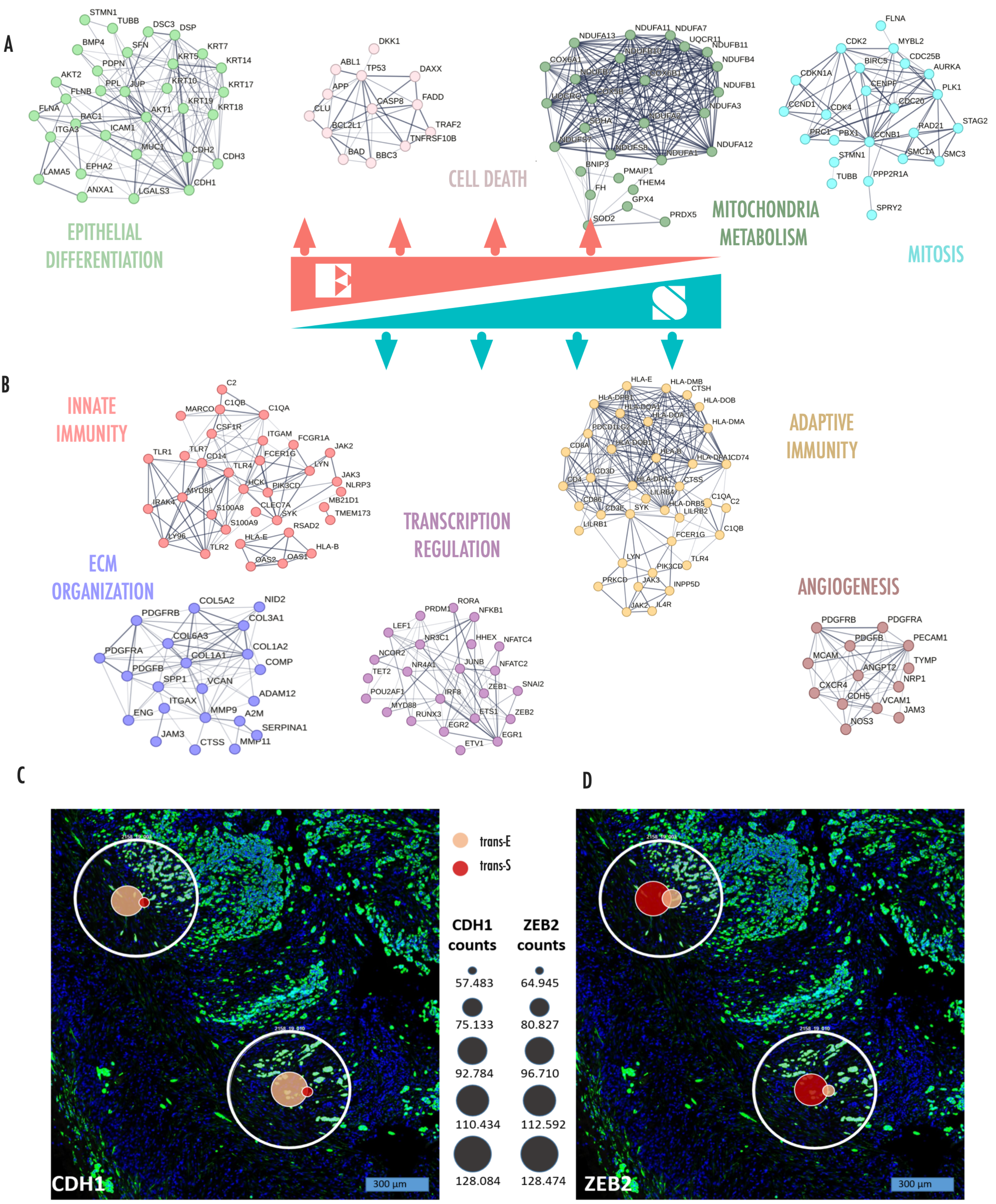

Supplementary FIGURE3

pS-AOIs vs pE-AOIs

A

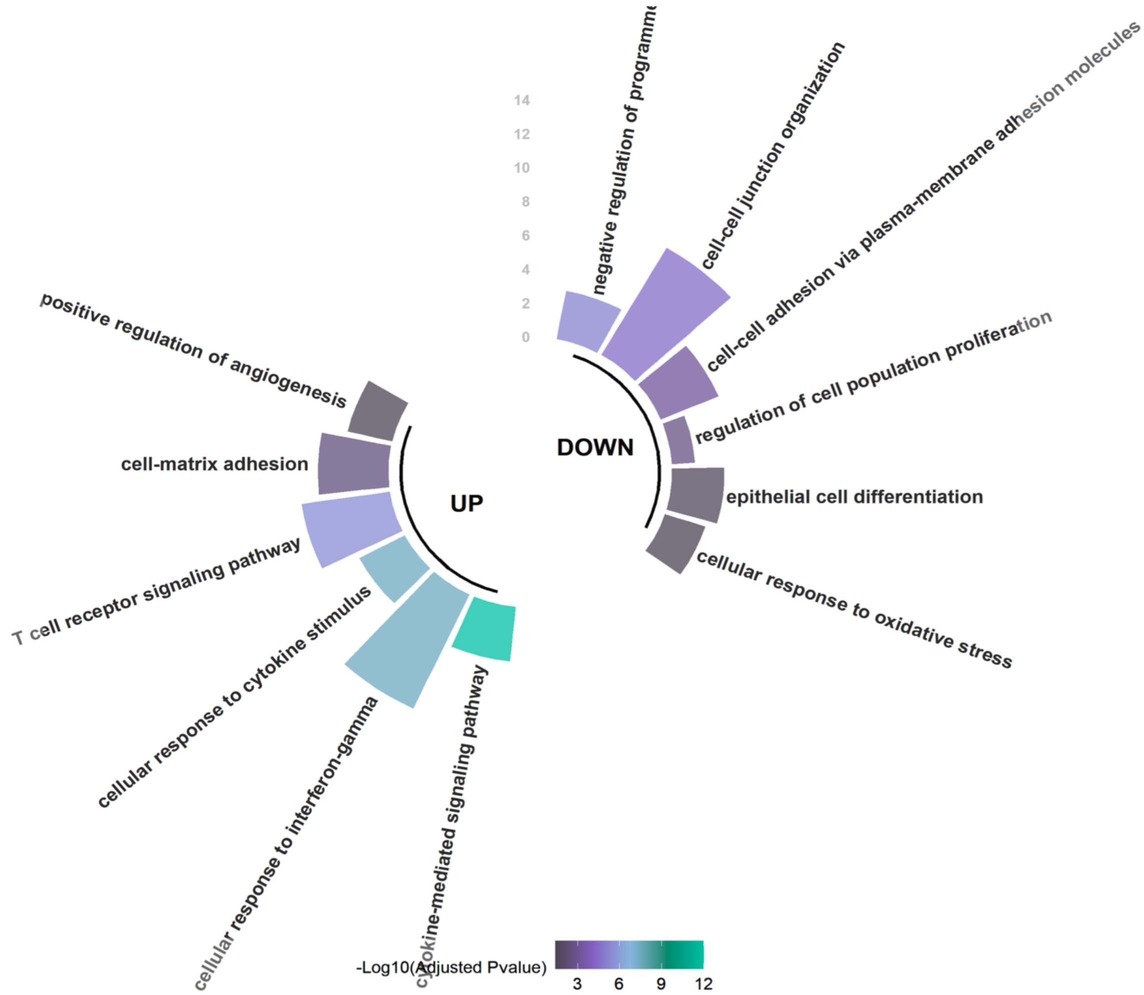

tS-AOIs vs tE-AOIs

B

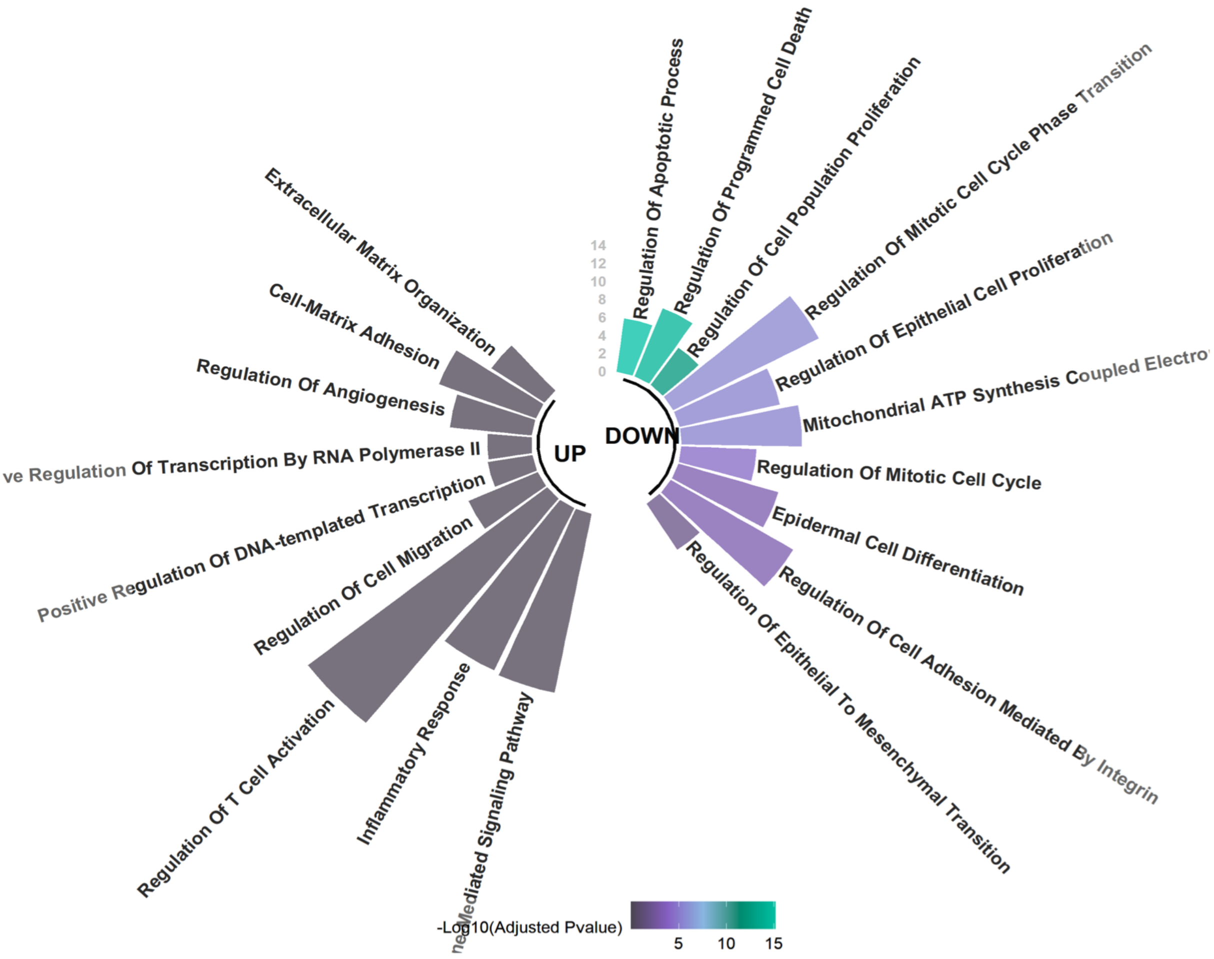

Supplementary Figure 4

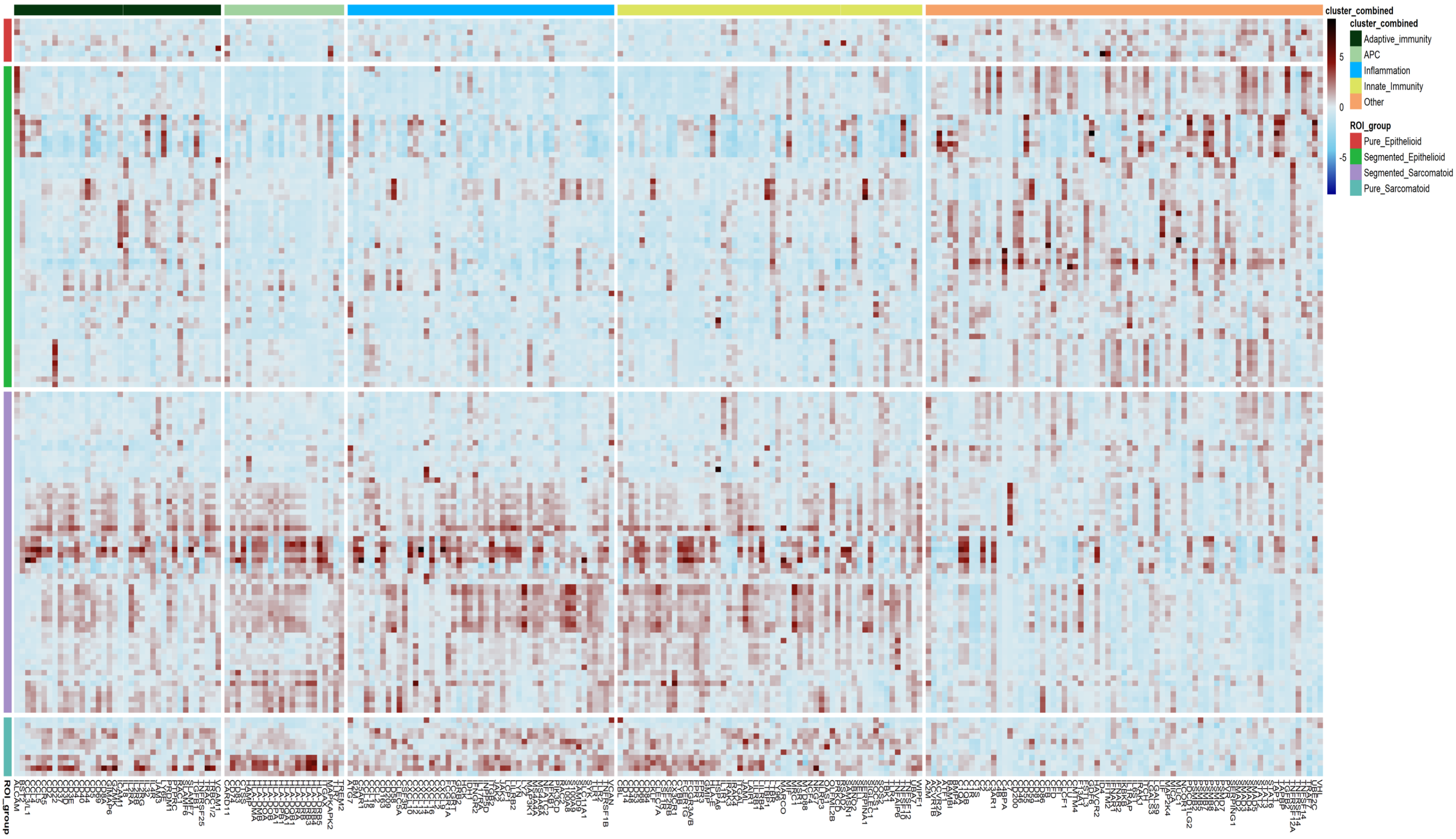

Supplementary Figure 5

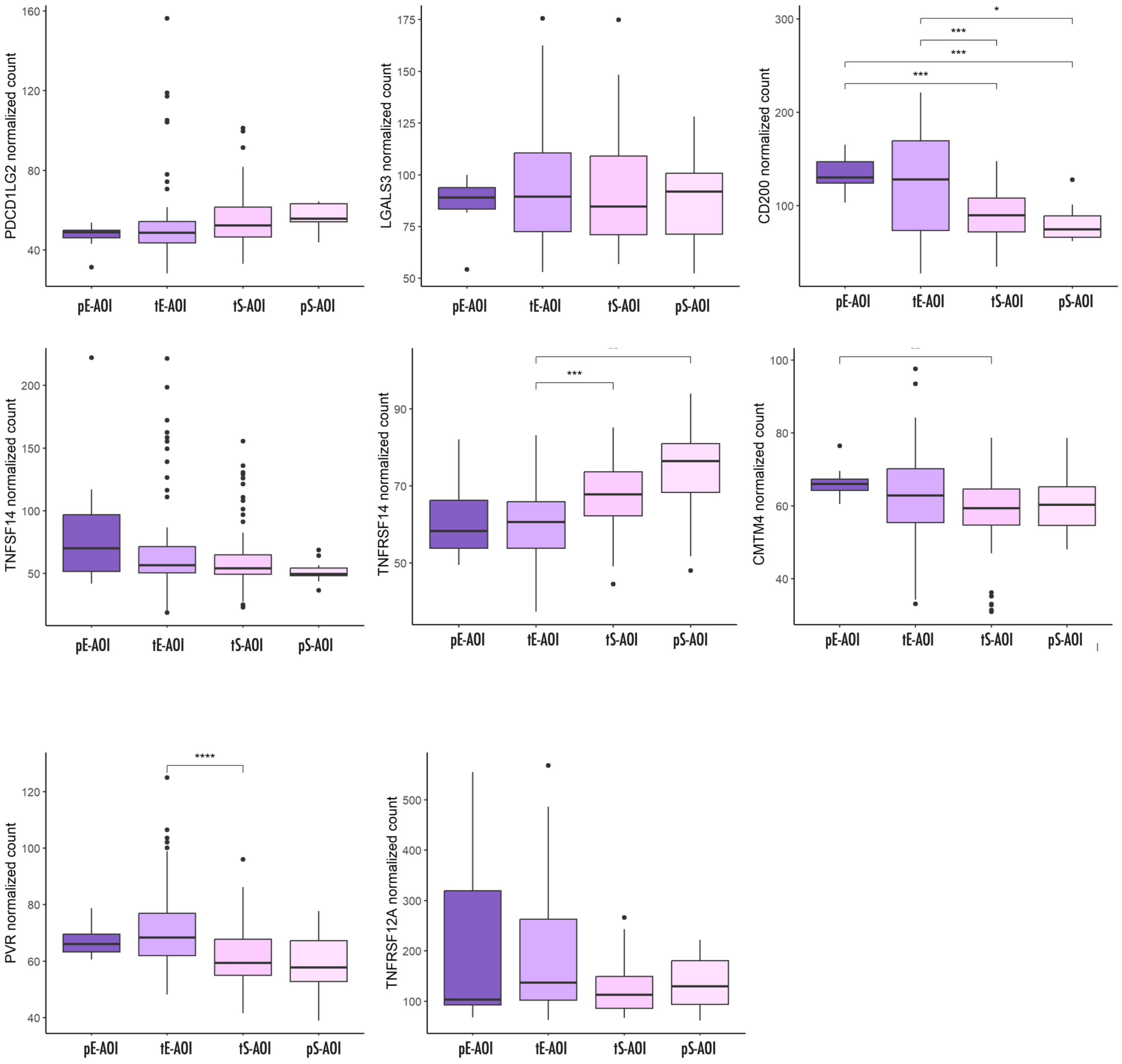

Supplementary Figure 6

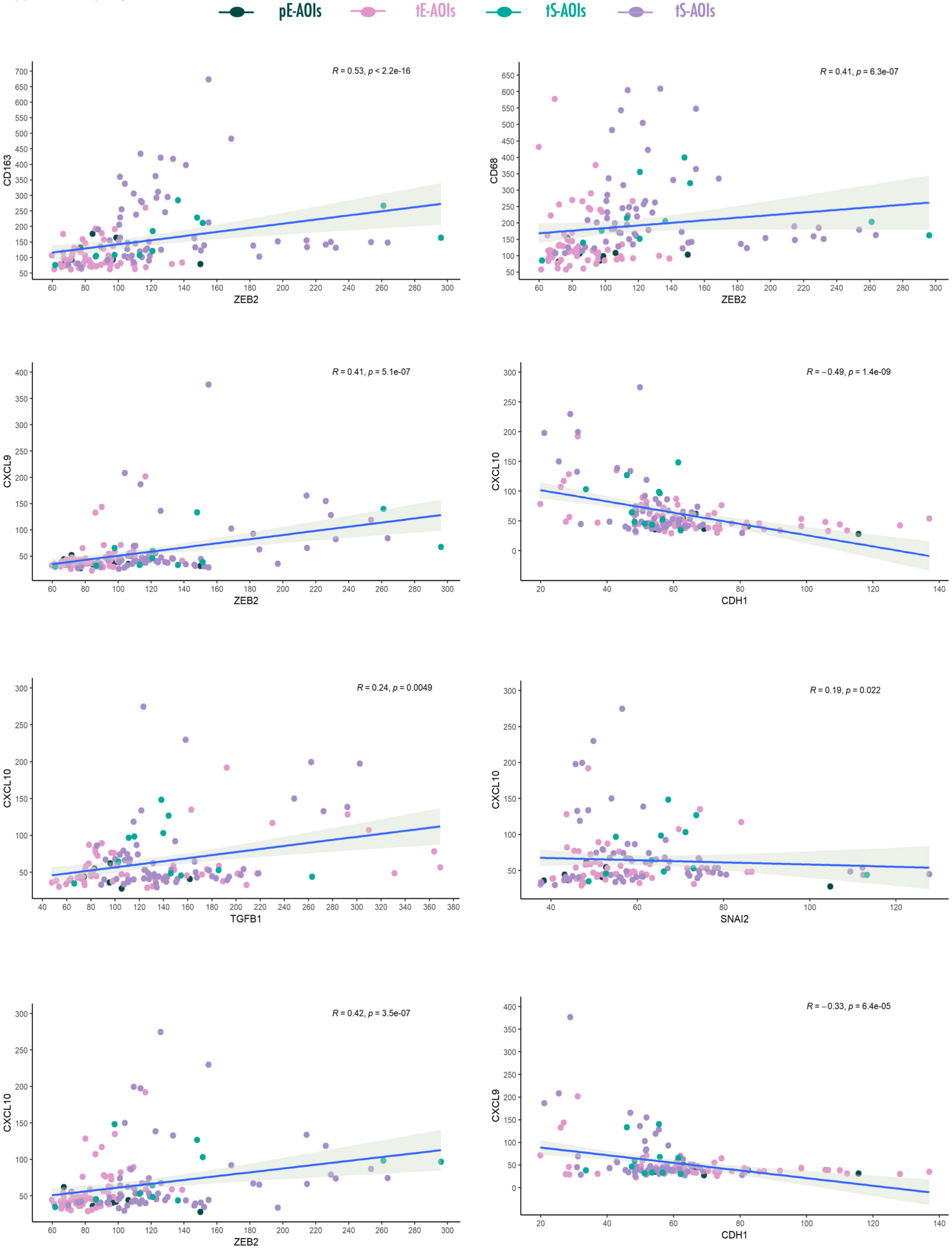

Supplementary Figure 7

LGASL9

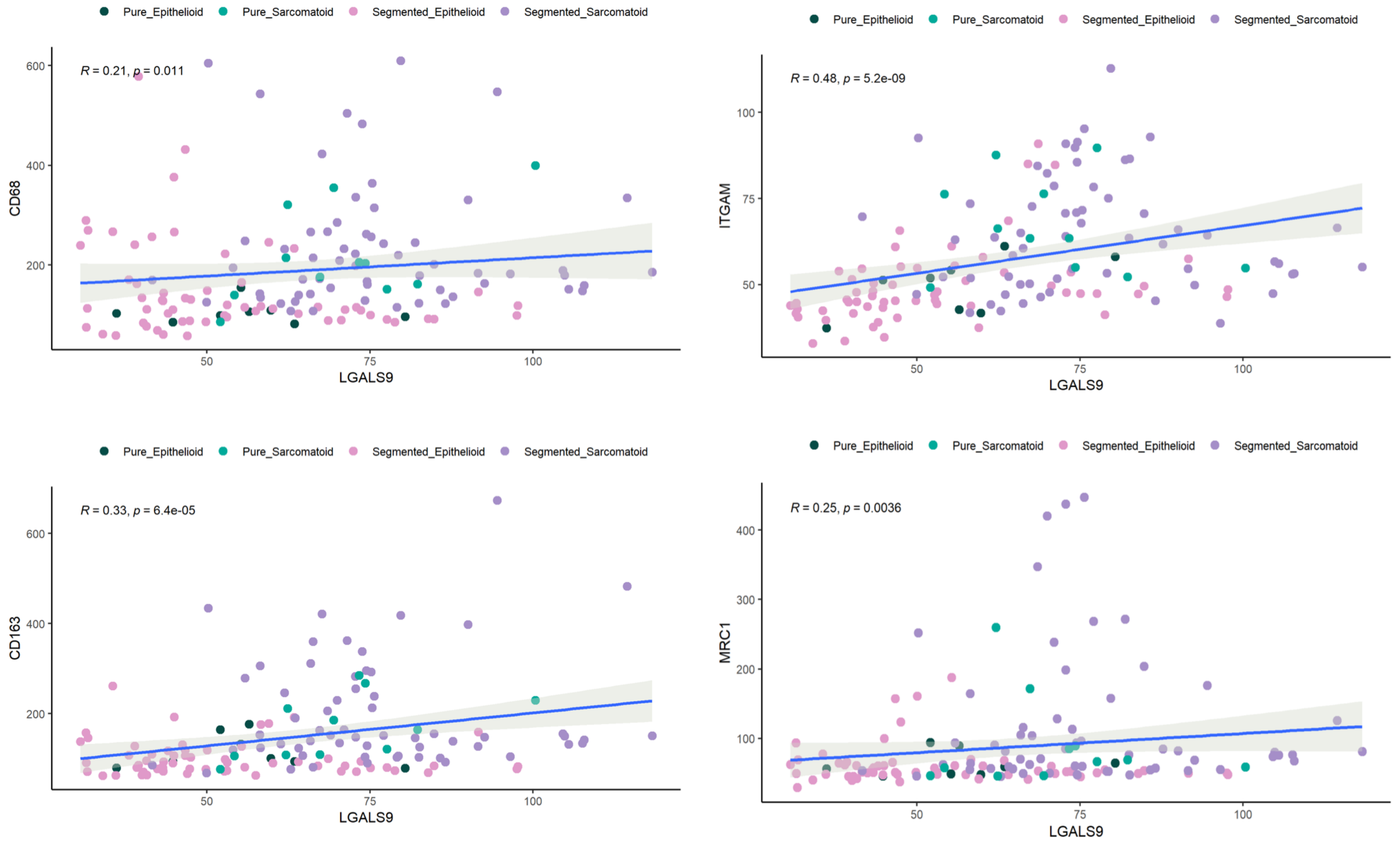

CD86

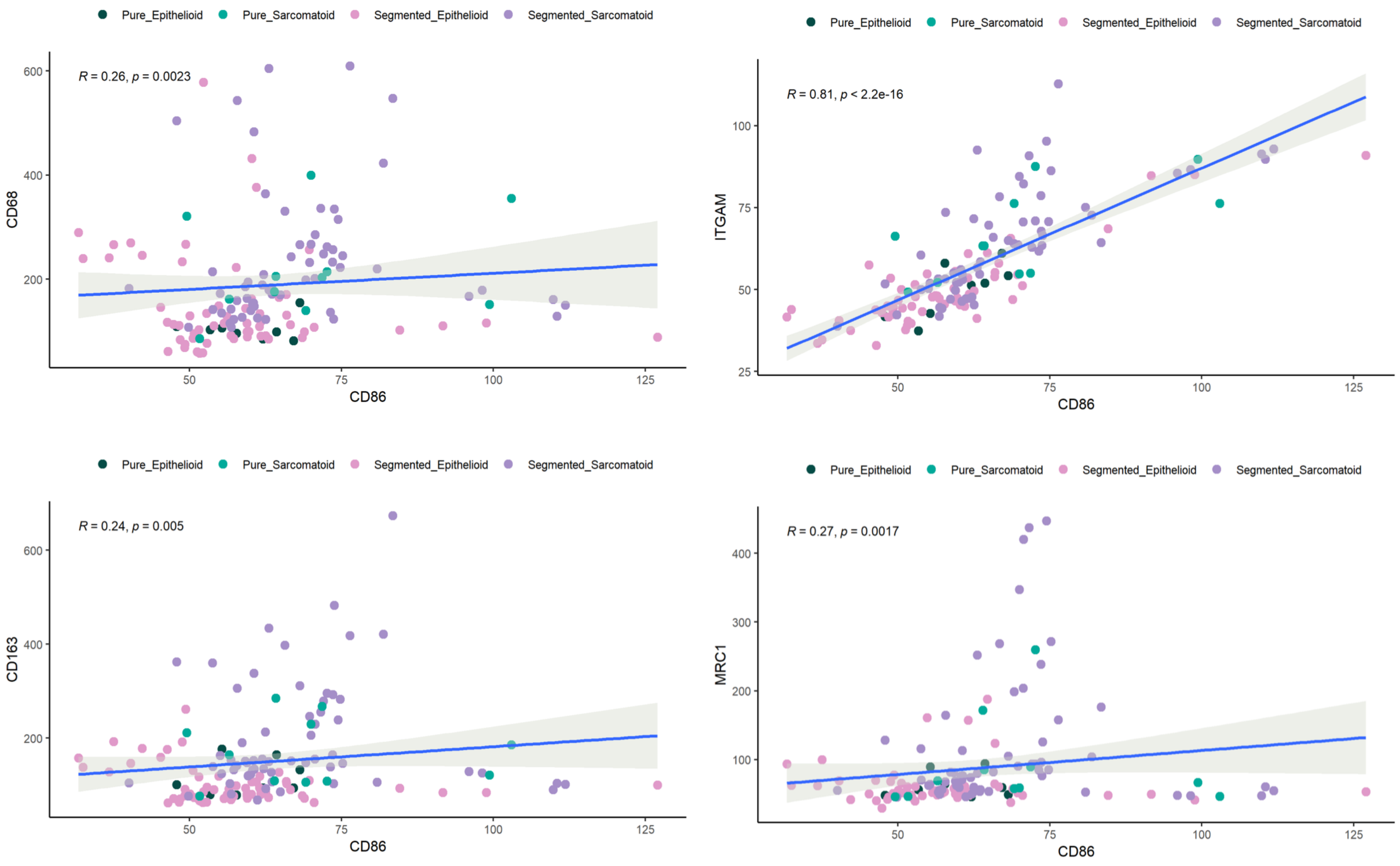

SUPPLEMENTARY FIGURE 8

A

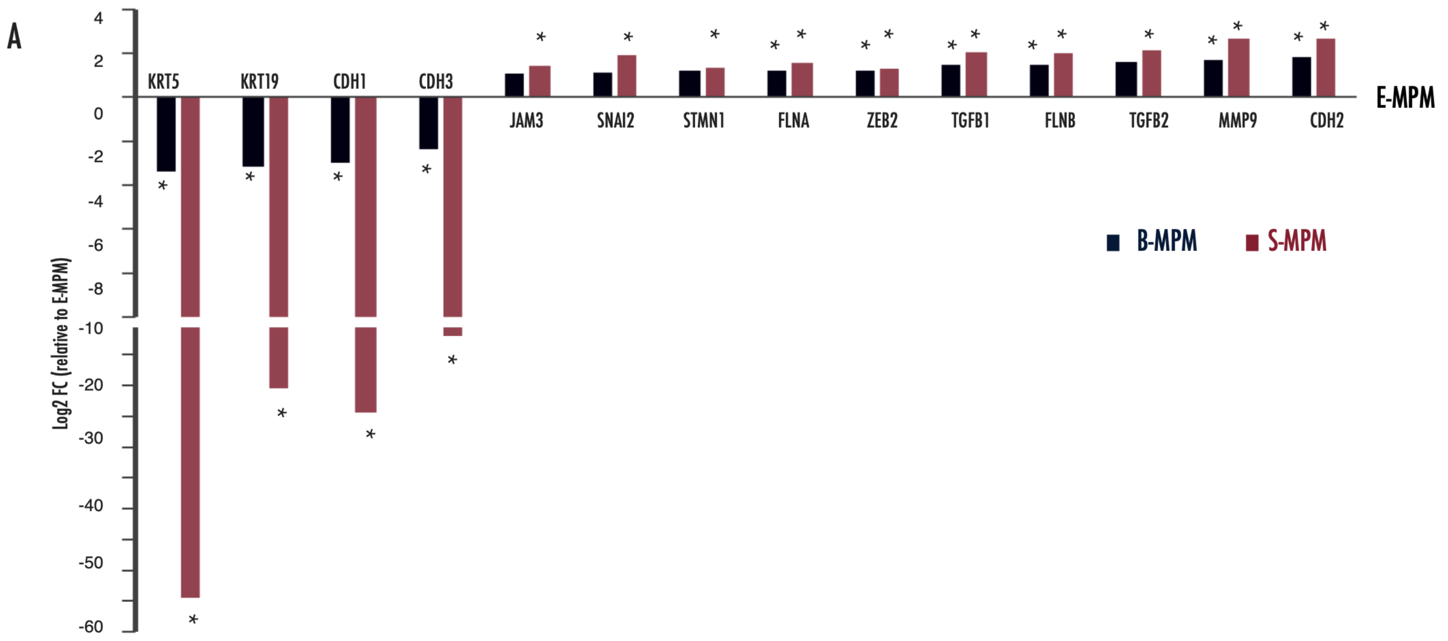

B

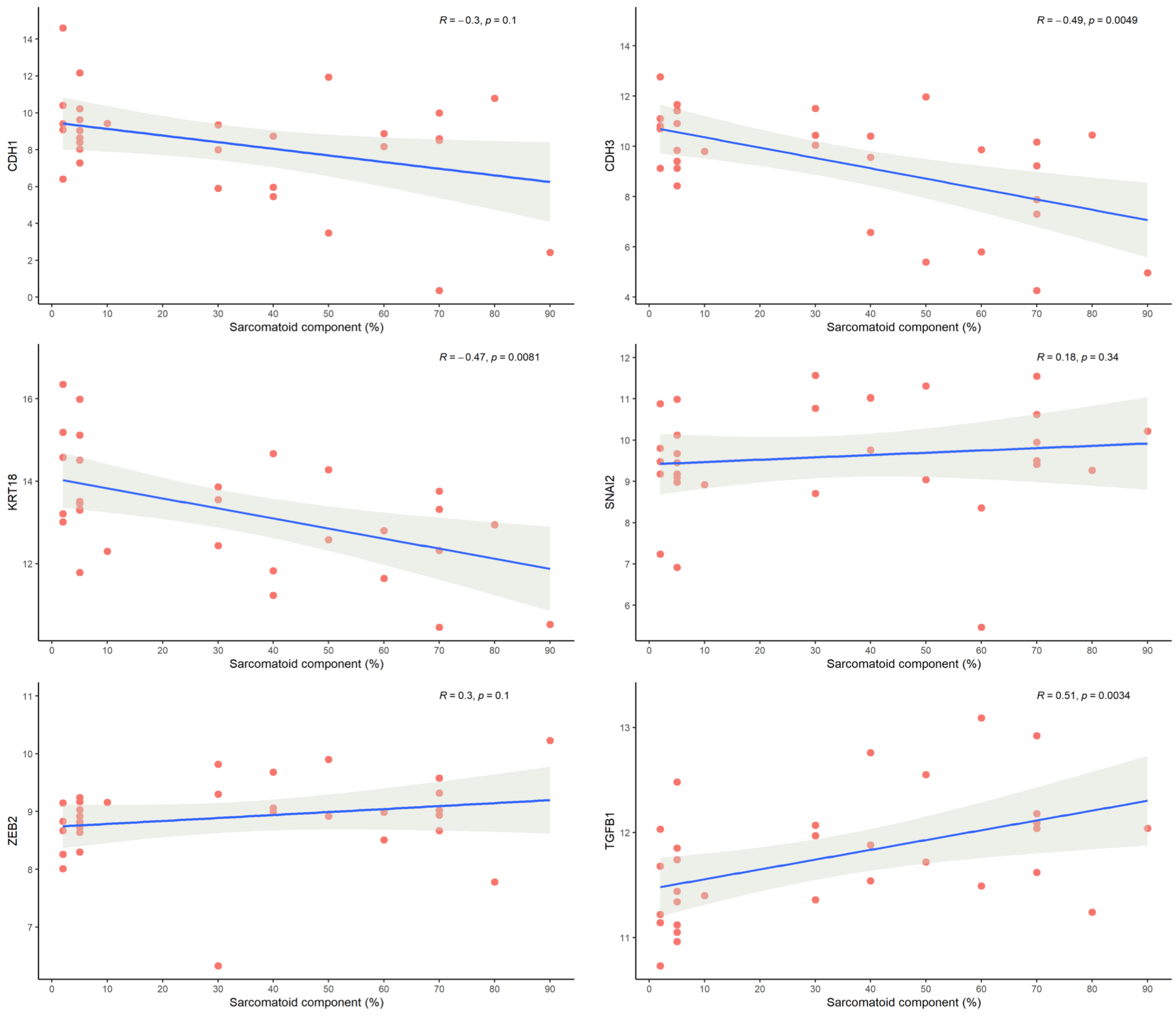

SUPPLEMENTARY FIGURE 9

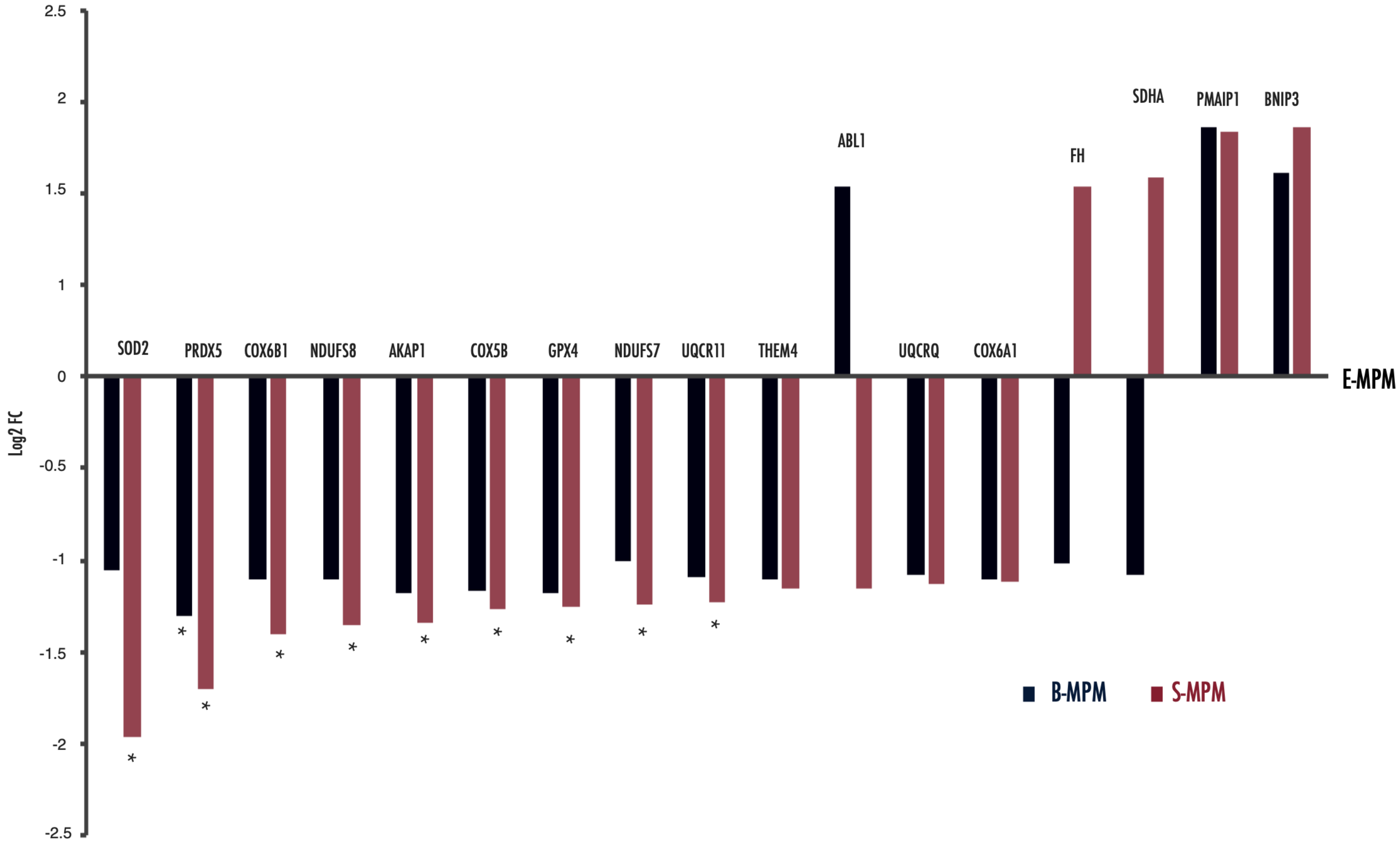

Supplementary Figure Legends

Supplementary Figure 1

A) GeoMx DSP scan showing AOIs from a representative B-MPM collected from a surgical pleurectomy. Large circles indicate segmented transitional AOIs while small circles circle indicate pure AOIs. B-C) Correlation analysis on all AOIs (B) or pure AOIs only (C)

Supplementary Figure 2

A-B) STRING interaction networks summarizing principal genes involved in the indicated GO categories and their association with either E (A) or S (B) compartment. The networks are representative of the categories emerged in the GO analysis reported in Figure 2C. C-D) Bubble plots showing expression of CDH1(C) and ZEB1 (D) in two representative transitional areas, showing the complementary of the signals.

Supplementary Figure 3

A) GO analysis on the genes differentially expressed between pS-AOIs and pE-AOIs. Full list of genes is provided in Supplementary Table 3. P value for GO categories is reported in Supplementary Table 4. B) GO analysis on the genes differentially expressed between tS-AOIs and tE-AOIs. Full list of genes is provided in Supplementary Table 5. P value for GO categories is reported in Supplementary Table 6.

Supplementary Figure 4

Heatmap representing expression of all deregulated immune related genes in the 4 AOI groups. Color gradient expresses the z-score of each gene in each sample.

Supplementary Figure 5

Box plots representing the expression level of indicated immune checkpoints in the different AOIs.

Supplementary Figure 6

Scatter plots showing the direct expression correlation of TAMs related markers with EMT TFs and markers across all AOIs.

Supplementary Figure 7

Scatter plots showing the direct expression correlation of TAMs related markers with immune checkpoints across all AOIs.

Supplementary Figure 8

A) Histograms reporting the relative expression of a panel of EMT markers in S-MPMs and biphasic MPMs (B-MPM) compared to E-MPMs. B) Scatter plots showing the correlation between the estimated percentage of sarcomatoid component and the expression of EMT markers in the B-MPMs subgroup included in our validation cohort.

Supplementary Figure 9

Histograms reporting the relative expression of a panel of genes involved in oxidative respiration and mitochondrial function in S-MPMs and biphasic MPMs (B-MPM) compared to E-MPMs.

Supplementary Table 1: Full list of differentially expressed genes in S vs E AOIs

Target name	*Log2 Fold Change (S-AOIs/E-AOIs)*	*Pvalue*	*Adjusted pvalue*
CSF1R	1.011133	1.92E-35	2.01E-32
A2M	1.263878	8.03E-35	4.21E-32
ITGA3	-1.18751	7.46E-33	2.02E-30
ENG	0.922176	7.69E-33	2.02E-30
FCER1G	0.835464	1.36E-32	2.85E-30
CTSS	0.892046	8.16E-32	1.43E-29
HLA-DMB	0.800581	1.08E-31	1.62E-29
FCGR3A/B	1.071155	2.61E-31	3.37E-29
CD4	0.769253	2.89E-31	3.37E-29
CD53	0.737308	1.06E-30	1.11E-28
C1QA	1.355796	2.16E-30	2.06E-28
C1QB	1.210335	4.63E-30	4.04E-28
PECAM1	0.813283	1.41E-29	1.06E-27
MS4A6A	0.834868	1.42E-29	1.06E-27
HLA-DMA	0.797812	3.62E-29	2.37E-27
ITGB2	0.711527	3.54E-29	2.37E-27
CD14	1.111031	7.34E-29	4.53E-27
HLA-DPA1	0.835945	1.48E-28	8.61E-27
LCP1	0.732087	2.02E-28	1.11E-26
HLA-DRB	1.43533	2.25E-28	1.18E-26
CYBB	0.830303	4.01E-28	2.00E-26
LAIR1	0.60908	7.92E-28	3.78E-26
FGCR2A/B	1.103551	1.95E-27	8.90E-26
WIPF1	0.498167	1.70E-26	7.43E-25
HLA-DRA	1.468951	2.02E-26	8.46E-25
TNFRSF1B	0.494245	4.26E-26	1.72E-24
HLA-DRB3	1.006682	8.68E-26	3.37E-24
PTPRC	0.724594	3.35E-25	1.26E-23
KRT18	-1.69168	4.42E-25	1.60E-23
LAMA5	-1.18119	8.77E-25	3.07E-23
GIMAP4	0.600613	1.37E-24	4.64E-23
CD74	1.655193	1.52E-24	4.99E-23
FCGR1A	0.642232	3.62E-24	1.15E-22
CD163	0.813758	4.30E-24	1.33E-22
IL10RA	0.576967	5.55E-24	1.66E-22
ZEB2	0.58503	7.83E-24	2.28E-22

HLA-DQA1	0.722306	8.96E-24	2.54E-22
KRT19	-1.68545	2.49E-23	6.87E-22
C3AR1	0.510641	3.47E-23	9.32E-22
LILRB4	0.598296	4.66E-23	1.22E-21
CLEC7A	0.560096	1.15E-22	2.95E-21
ITGB4	-0.93849	2.76E-22	6.74E-21
LYZ	1.341299	2.75E-22	6.74E-21
TBXAS1	0.489776	3.48E-22	8.29E-21
CD84	0.407021	6.26E-22	1.46E-20
FPR3	0.402665	8.04E-22	1.83E-20
LAMC2	-0.7411	1.56E-21	3.49E-20
EPHA2	-0.54279	2.05E-21	4.47E-20
PRKDC	-0.43854	3.59E-21	7.69E-20
CD37	0.446635	5.69E-21	1.19E-19
SPRED1	0.341705	9.36E-21	1.92E-19
SLC39A6	-0.27555	1.69E-20	3.40E-19
CXCL16	0.392065	2.72E-20	5.38E-19
MS4A4A	0.505259	3.05E-20	5.93E-19
CFB	-1.28994	4.31E-20	8.21E-19
NCF4	0.423397	5.03E-20	9.42E-19
HLA-DOA	0.50412	5.20E-20	9.57E-19
CDH2	-0.70999	8.03E-20	1.45E-18
HLA-DRB4	0.812502	1.03E-19	1.80E-18
MET	-0.73598	1.01E-19	1.80E-18
IGF1R	-0.51112	1.13E-19	1.95E-18
CXADR	-0.4737	1.18E-19	1.99E-18
PPL	-0.65079	1.65E-19	2.75E-18
TLR1	0.365261	1.76E-19	2.88E-18
HCK	0.376192	5.39E-19	8.69E-18
INPP5D	0.430494	6.72E-19	1.07E-17
LGALS9	0.524902	6.90E-19	1.08E-17
DSP	-0.72458	2.33E-18	3.60E-17
FSTL3	-0.61115	9.79E-18	1.49E-16
FZD6	-0.35898	1.21E-17	1.81E-16
MRC1	0.627983	1.36E-17	1.98E-16
CD68	0.727373	1.34E-17	1.98E-16
FPR1	0.362439	1.84E-17	2.64E-16
CD9	-0.70204	2.00E-17	2.83E-16
FGF9	-0.53115	3.79E-17	5.30E-16
JAK3	0.401846	4.69E-17	6.47E-16
GIMAP6	0.391801	5.28E-17	7.19E-16
FLNC	-0.77458	8.01E-17	1.08E-15
CD34	0.451966	1.55E-16	2.06E-15
HAVCR2	0.340373	2.13E-16	2.79E-15

COL6A3	1.332912	2.24E-16	2.89E-15
TLR2	0.430028	2.90E-16	3.67E-15
SAMSN1	0.391422	2.87E-16	3.67E-15
ITGA1	0.596449	5.54E-16	6.92E-15
PDGFRB	0.77377	6.07E-16	7.49E-15
ITGA4	0.348064	6.37E-16	7.77E-15
CXCL12	0.547031	6.79E-16	8.19E-15
TP53	-0.36318	7.77E-16	9.26E-15
CSF3R	0.363416	9.98E-16	1.18E-14
COX6A1	-0.41151	1.44E-15	1.68E-14
GMIP	0.241928	2.12E-15	2.44E-14
SMARCA4	-0.36585	3.38E-15	3.85E-14
PTGER4	0.288235	6.46E-15	7.29E-14
IRF8	0.431974	8.44E-15	9.42E-14
TNFSF12	0.292745	8.70E-15	9.60E-14
HSP90B1	-0.56841	9.01E-15	9.84E-14
GPSM3	0.29494	1.01E-14	1.09E-13
SYK	0.319652	1.24E-14	1.33E-13
THY1	0.61562	1.36E-14	1.44E-13
MUC1	-0.55425	1.63E-14	1.71E-13
SIGLEC1	0.42458	1.70E-14	1.77E-13
LY6E	-0.61207	2.09E-14	2.15E-13
ITGAX	0.390248	2.57E-14	2.62E-13
NID2	0.46234	3.43E-14	3.46E-13
MSR1	0.400176	3.65E-14	3.64E-13
GLS	-0.66658	5.84E-14	5.78E-13
EFNA1	-0.4241	7.92E-14	7.77E-13
SKP2	-0.36431	9.21E-14	8.94E-13
RASA4	0.292567	9.59E-14	9.15E-13
CAPN2	-0.41913	9.58E-14	9.15E-13
S100A9	0.682518	1.49E-13	1.41E-12
F13A1	0.811226	1.68E-13	1.57E-12
ACTA2	0.733924	1.91E-13	1.77E-12
PVR	-0.21645	2.70E-13	2.48E-12
SFRP4	0.657073	2.96E-13	2.70E-12
C4B	-1.31006	3.00E-13	2.71E-12
JAML	0.445249	3.47E-13	3.11E-12
PRDX5	-0.328	4.48E-13	3.98E-12
EGFR	-0.56388	7.19E-13	6.28E-12
LILRB1	0.315144	7.15E-13	6.28E-12
TFRC	-0.38001	7.83E-13	6.79E-12
RAC2	0.390937	9.66E-13	8.31E-12
TLR4	0.312615	1.04E-12	8.90E-12
ERBB2	-0.32928	1.16E-12	9.78E-12

CDH3	-0.40994	1.52E-12	1.28E-11
PRDM1	0.291707	2.25E-12	1.87E-11
ITGB8	-0.28415	2.34E-12	1.93E-11
EFNA5	-0.37854	3.10E-12	2.54E-11
ELMO1	0.315471	3.21E-12	2.61E-11
HLA-DQB1	0.348572	3.95E-12	3.19E-11
C1S	-0.73027	4.19E-12	3.35E-11
ARNT2	-0.21299	4.50E-12	3.58E-11
ITGAL	0.374261	4.63E-12	3.65E-11
ATP2A2	-0.37016	4.98E-12	3.90E-11
WEE1	-0.29392	5.03E-12	3.91E-11
SFRP2	0.773609	5.08E-12	3.92E-11
TREM2	0.304756	5.16E-12	3.95E-11
YTHDF2	-0.21862	6.63E-12	5.04E-11
ILF3	-0.3736	6.96E-12	5.25E-11
TNFRSF10B	-0.25682	7.79E-12	5.83E-11
SLC16A1	-0.54001	7.92E-12	5.89E-11
NOTCH2	-0.47509	8.59E-12	6.35E-11
MERTK	0.265167	8.89E-12	6.52E-11
ITGAM	0.379229	9.34E-12	6.76E-11
LILRB2	0.468208	9.31E-12	6.76E-11
CD46	-0.24684	1.48E-11	1.06E-10
MCAM	0.499208	1.80E-11	1.29E-10
PLOD2	-0.85263	1.93E-11	1.37E-10
NDUFA13	-0.33889	2.00E-11	1.41E-10
TPR	-0.23764	2.09E-11	1.46E-10
LYN	0.223166	2.53E-11	1.76E-10
API5	-0.19476	2.61E-11	1.80E-10
NRP1	0.54055	3.25E-11	2.23E-10
WT1	-0.48921	3.90E-11	2.66E-10
KRT5	-0.64177	4.48E-11	3.03E-10
GAS1	-0.69393	4.79E-11	3.22E-10
MST1R	-0.31824	5.18E-11	3.46E-10
RAD21	-0.39823	5.22E-11	3.47E-10
NECTIN2	-0.47488	5.80E-11	3.82E-10
C5AR1	0.287622	7.39E-11	4.85E-10
NDUFS8	-0.2476	8.24E-11	5.37E-10
PSMB10	0.239819	8.43E-11	5.46E-10
EIF4EBP1	-0.29937	9.06E-11	5.83E-10
IL2RB	0.327839	9.30E-11	5.95E-10
NUP107	-0.20811	9.96E-11	6.33E-10
PUM1	-0.23473	1.01E-10	6.35E-10
TRAC	0.436342	1.04E-10	6.56E-10
MIF	-0.57136	1.27E-10	7.95E-10

CDH5	0.34026	1.32E-10	8.22E-10
OLFML2B	0.421618	1.39E-10	8.60E-10
LIF	-0.33737	1.47E-10	8.99E-10
NDUFA7	-0.19868	1.66E-10	1.01E-09
CLU	-0.85908	1.80E-10	1.09E-09
DSC3	-0.25615	1.87E-10	1.13E-09
GTF3C1	-0.25896	1.91E-10	1.15E-09
ALCAM	-0.57583	2.05E-10	1.22E-09
SERINC3	-0.18859	2.11E-10	1.25E-09
PSMB7	-0.32828	2.29E-10	1.35E-09
STAG2	-0.17272	2.38E-10	1.40E-09
C7	0.851285	2.50E-10	1.46E-09
ASNS	-0.24815	2.86E-10	1.66E-09
TNFRSF1A	-0.26952	3.60E-10	2.07E-09
CCL3/L1	0.40565	3.78E-10	2.17E-09
CACNB3	-0.28114	4.37E-10	2.49E-09
CD36	0.354452	4.83E-10	2.74E-09
SIN3A	-0.14567	5.40E-10	3.04E-09
VCAN	0.755108	6.23E-10	3.50E-09
KEAP1	-0.16498	6.68E-10	3.73E-09
SOD1	-0.2851	7.24E-10	4.02E-09
MAP3K7	-0.19139	7.89E-10	4.35E-09
NDUFB10	-0.19283	9.94E-10	5.43E-09
AXIN1	-0.19317	9.93E-10	5.43E-09
MSH6	-0.19899	1.13E-09	6.15E-09
GOT2	-0.16908	1.14E-09	6.15E-09
MCM7	-0.28625	1.18E-09	6.37E-09
RIPK3	0.204609	1.21E-09	6.47E-09
FLNB	-0.40747	1.22E-09	6.49E-09
IL4R	0.171963	1.36E-09	7.22E-09
PPP2R1A	-0.28259	1.42E-09	7.48E-09
CD200	-0.39922	1.47E-09	7.71E-09
BMP4	-0.41149	1.61E-09	8.38E-09
CIC	-0.17645	1.61E-09	8.38E-09
FCGRT	0.288929	1.69E-09	8.68E-09
C1R	-0.55416	1.69E-09	8.68E-09
COL3A1	0.607205	1.77E-09	9.06E-09
SLC11A1	0.330345	1.93E-09	9.85E-09
CMKLR1	0.297513	3.01E-09	1.53E-08
ANLN	-0.25485	3.08E-09	1.55E-08
CDK6	-0.48132	3.23E-09	1.62E-08
DNAJC14	-0.15028	3.45E-09	1.73E-08
MAP3K20	-0.25984	3.90E-09	1.94E-08
TNFRSF12A	-0.36664	4.05E-09	2.01E-08

REL	0.211566	4.45E-09	2.19E-08
TLR7	0.254655	4.53E-09	2.22E-08
PRKCD	0.170845	5.15E-09	2.51E-08
MCM4	-0.22748	5.23E-09	2.54E-08
BBS1	-0.1559	5.83E-09	2.82E-08
APP	-0.53077	6.40E-09	3.08E-08
NDUFA1	-0.20942	6.46E-09	3.10E-08
SMAD3	-0.19904	6.57E-09	3.13E-08
IL17RA	0.215204	6.72E-09	3.19E-08
ANXA1	-0.37111	6.92E-09	3.27E-08
BAD	-0.19519	7.66E-09	3.60E-08
HSPA6	0.513136	8.05E-09	3.77E-08
C1QBP	-0.22892	8.50E-09	3.96E-08
IFNGR2	0.262482	1.08E-08	5.01E-08
TYMP	0.332293	1.18E-08	5.47E-08
NUMBL	-0.24598	1.26E-08	5.80E-08
COL1A2	0.696029	1.30E-08	5.95E-08
FLT1	0.309169	1.35E-08	6.18E-08
CSF2RB	0.252021	1.56E-08	7.10E-08
PRKAR1B	-0.24644	1.58E-08	7.16E-08
CASP1	0.239581	1.74E-08	7.83E-08
PSMC4	-0.13169	1.84E-08	8.26E-08
PDGFC	-0.22369	1.93E-08	8.63E-08
STMN1	-0.23648	2.00E-08	8.87E-08
IL2RA	0.292979	2.09E-08	9.27E-08
ETS1	0.261692	2.27E-08	1.00E-07
APLNR	0.288806	2.44E-08	1.07E-07
TNKS	-0.13662	2.50E-08	1.09E-07
COX6B1	-0.24415	2.58E-08	1.12E-07
HLA-DRB5	0.237889	2.58E-08	1.12E-07
TUBB	-0.4146	2.63E-08	1.13E-07
IL2RG	0.293012	2.70E-08	1.16E-07
TGFB2	-0.7393	2.73E-08	1.17E-07
PHGDH	-0.30327	2.75E-08	1.17E-07
TRBC1/2	0.545807	3.00E-08	1.28E-07
HDAC6	-0.13761	3.08E-08	1.30E-07
FGF18	-0.29148	3.42E-08	1.44E-07
VCAM1	0.289027	3.50E-08	1.47E-07
IL21R	0.231736	3.74E-08	1.56E-07
POLR2H	-0.18683	3.81E-08	1.58E-07
AKT2	-0.22174	4.14E-08	1.72E-07
PIK3CD	0.198624	4.39E-08	1.81E-07
IL1R1	0.231761	5.48E-08	2.24E-07
FGFR1	-0.3553	5.46E-08	2.24E-07

RRAS2	-0.1975	5.48E-08	2.24E-07
SERINC1	-0.21611	5.78E-08	2.35E-07
CCNB1	-0.21688	5.80E-08	2.35E-07
SMC1A	-0.14292	6.16E-08	2.49E-07
NDUFB7	-0.1337	6.57E-08	2.64E-07
CD48	0.239847	6.78E-08	2.72E-07
THEM4	-0.14663	7.02E-08	2.80E-07
ITGA6	-0.17314	7.56E-08	3.00E-07
NDUFA11	-0.23717	7.80E-08	3.09E-07
SMPD3	-0.49242	7.98E-08	3.15E-07
NKG7	0.29034	8.31E-08	3.26E-07
STAT3	-0.29646	8.44E-08	3.30E-07
FOXC1	-0.21379	8.95E-08	3.49E-07
CKLF	0.242249	9.31E-08	3.61E-07
WNT2B	-0.52325	9.31E-08	3.61E-07
ICAM1	-0.44791	9.88E-08	3.81E-07
HACD2	-0.24299	1.01E-07	3.89E-07
UQCR11	-0.11729	1.06E-07	4.05E-07
CNOT2	-0.12343	1.32E-07	5.02E-07
CCL4	0.358323	1.33E-07	5.05E-07
S100A8	0.394993	1.36E-07	5.15E-07
RPL23	-0.40287	1.64E-07	6.15E-07
U2AF1	-0.24714	1.64E-07	6.15E-07
MDM2	-0.31399	1.73E-07	6.42E-07
CX3CR1	0.347282	1.73E-07	6.42E-07
TMEM140	0.209458	1.94E-07	7.21E-07
KRT7	-0.56868	2.01E-07	7.43E-07
PPP2CB	-0.23075	2.09E-07	7.70E-07
CDK4	-0.21753	2.30E-07	8.44E-07
TOP2A	-0.32738	2.33E-07	8.50E-07
CD55	-0.20731	2.39E-07	8.69E-07
NDUFB4	-0.18874	2.51E-07	9.12E-07
ANP32B	-0.24397	2.76E-07	9.97E-07
SFXN1	-0.1617	2.77E-07	9.97E-07
CCL5	0.390667	2.93E-07	1.05E-06
CD2	0.282315	2.96E-07	1.06E-06
TRAF7	-0.16929	3.05E-07	1.09E-06
RUNX3	0.221669	3.32E-07	1.18E-06
ITCH	-0.20451	3.35E-07	1.19E-06
RB1	0.128938	3.45E-07	1.22E-06
ERCC3	-0.14377	3.71E-07	1.31E-06
COX5B	-0.21632	3.90E-07	1.37E-06
PLA2G2A	-0.5338	4.19E-07	1.47E-06
CTSH	0.306831	4.73E-07	1.64E-06

MAPK9	-0.10472	4.72E-07	1.64E-06
IL18	-0.38205	4.92E-07	1.70E-06
LDHB	-0.33068	4.99E-07	1.72E-06
CD83	0.232294	5.31E-07	1.82E-06
ANKRD28	-0.18854	5.78E-07	1.98E-06
MSH2	-0.14059	5.80E-07	1.98E-06
UBA7	0.17068	6.55E-07	2.22E-06
MAP3K1	0.164434	6.60E-07	2.23E-06
PSMB2	-0.10063	7.39E-07	2.49E-06
COL1A1	0.52775	7.48E-07	2.51E-06
B2M	0.508611	8.07E-07	2.71E-06
ERCC2	-0.16073	8.20E-07	2.74E-06
RAF1	-0.13711	8.56E-07	2.85E-06
BST2	-0.22846	8.68E-07	2.88E-06
NPM1	-0.19704	9.75E-07	3.23E-06
GNA11	-0.14911	1.05E-06	3.46E-06
PTPN11	-0.14803	1.07E-06	3.50E-06
CXCR4	0.459678	1.08E-06	3.53E-06
PEBP1	-0.28578	1.09E-06	3.56E-06
NF1	-0.17544	1.18E-06	3.83E-06
MRPL19	-0.14359	1.26E-06	4.09E-06
MEN1	-0.15629	1.39E-06	4.49E-06
AKAP1	-0.14987	1.60E-06	5.17E-06
RAC1	-0.23297	1.78E-06	5.74E-06
NDUFA12	-0.10921	1.82E-06	5.84E-06
CCR5	0.229333	1.94E-06	6.21E-06
BRD3	-0.21992	2.12E-06	6.77E-06
CDC25B	-0.24738	2.25E-06	7.12E-06
HDAC2	-0.20922	2.24E-06	7.12E-06
CDH1	-0.26031	2.44E-06	7.70E-06
SFN	-0.2068	2.48E-06	7.81E-06
TRAF2	-0.13944	2.89E-06	9.06E-06
IL1RAP	-0.21141	2.90E-06	9.07E-06
CD47	-0.19401	2.91E-06	9.09E-06
NSD3	-0.15501	3.05E-06	9.48E-06
NLRC5	0.187998	3.21E-06	9.95E-06
SRSF2	-0.20186	3.31E-06	1.02E-05
CD86	0.246606	3.35E-06	1.03E-05
MFNG	0.16072	3.37E-06	1.04E-05
GNG12	-0.14149	3.40E-06	1.04E-05
EGR2	0.25951	3.45E-06	1.06E-05
BCL2	0.195452	3.60E-06	1.10E-05
ACY1	-0.14471	3.62E-06	1.10E-05
SMC3	-0.1106	3.70E-06	1.12E-05

CENPF	-0.20534	4.11E-06	1.24E-05
FLNA	-0.35701	4.57E-06	1.38E-05
SAA1	-0.61135	4.78E-06	1.44E-05
SDHA	-0.17386	4.94E-06	1.48E-05
SMAD4	-0.15137	5.19E-06	1.55E-05
TAPBP	-0.12957	5.33E-06	1.59E-05
ANGPT2	0.223532	5.40E-06	1.60E-05
CCND2	0.239758	5.68E-06	1.68E-05
FANCF	-0.125	5.92E-06	1.75E-05
CTSW	0.197295	5.96E-06	1.76E-05
CACNG4	-0.1854	5.99E-06	1.76E-05
PDPN	-0.33676	6.51E-06	1.91E-05
POLR2A	-0.17203	6.97E-06	2.04E-05
ARNT	-0.13812	7.09E-06	2.07E-05
COL4A5	-0.27425	7.21E-06	2.09E-05
POLR2D	-0.12765	7.22E-06	2.09E-05
H2AX	-0.20398	7.36E-06	2.13E-05
LTBR	-0.16592	7.49E-06	2.16E-05
MIB1	-0.11281	9.17E-06	2.63E-05
C2	0.214297	9.45E-06	2.70E-05
MYC	-0.32905	9.81E-06	2.80E-05
NFATC2	0.16967	1.01E-05	2.88E-05
PCNA	-0.19601	1.12E-05	3.17E-05
GTF2H3	-0.10131	1.16E-05	3.29E-05
SOS2	0.112683	1.20E-05	3.38E-05
RAD50	-0.11461	1.25E-05	3.51E-05
JUP	-0.14144	1.28E-05	3.59E-05
DIPK2B	0.242099	1.34E-05	3.75E-05
ALDOA	-0.41327	1.64E-05	4.57E-05
ADAM12	0.201032	1.69E-05	4.69E-05
TNFRSF14	0.164197	1.78E-05	4.95E-05
MAVS	-0.13468	1.86E-05	5.16E-05
JAM3	0.136364	1.94E-05	5.36E-05
JAK2	0.153036	1.96E-05	5.41E-05
CPSF7	-0.16164	2.08E-05	5.72E-05
CD3E	0.221651	2.17E-05	5.94E-05
MAP2K2	-0.17644	2.28E-05	6.23E-05
NFE2L2	-0.16535	2.34E-05	6.38E-05
NOTCH3	0.417147	2.39E-05	6.48E-05
TFE3	-0.13268	2.49E-05	6.76E-05
NSD2	-0.13151	2.58E-05	6.96E-05
TET2	0.127417	2.67E-05	7.17E-05
BAMBI	-0.15289	2.66E-05	7.17E-05
PDGFB	0.199203	2.67E-05	7.17E-05

RRAD	-0.25499	2.85E-05	7.64E-05
UBC	-0.27498	3.00E-05	8.00E-05
ABCF1	-0.12127	3.10E-05	8.26E-05
RICTOR	-0.10823	3.24E-05	8.60E-05
NDUFA3	-0.11417	3.56E-05	9.42E-05
BRD4	-0.22659	3.62E-05	9.57E-05
TBP	-0.10672	3.68E-05	9.70E-05
FUBP1	-0.16627	3.75E-05	9.85E-05
CD81	-0.32966	3.90E-05	0.000102
IRAK1	-0.21545	3.95E-05	0.000103
RPL7A	-0.42034	4.11E-05	0.000107
LEF1	0.23489	4.22E-05	0.000109
CCND1	-0.26113	4.37E-05	0.000113
CD8A	0.206745	4.38E-05	0.000113
HHIP	-0.44276	4.41E-05	0.000114
ATF1	-0.10827	4.51E-05	0.000116
C4BPA	-0.27924	4.57E-05	0.000117
IL6ST	-0.2142	4.74E-05	0.000121
STK11	-0.14036	4.75E-05	0.000121
STING1	0.122967	4.73E-05	0.000121
ATP5ME	-0.11196	4.75E-05	0.000121
TNFRSF11B	-0.17061	4.79E-05	0.000121
MAP3K12	-0.14819	4.86E-05	0.000123
CACNA1C	0.16008	5.05E-05	0.000127
GPI	-0.39912	5.38E-05	0.000135
IDH2	0.103414	5.93E-05	0.000149
GBP4	0.210449	6.03E-05	0.000151
TFDP1	-0.11404	6.04E-05	0.000151
LFNG	0.236907	6.09E-05	0.000151
BCL6	-0.13044	6.25E-05	0.000155
CDK2	-0.13179	6.45E-05	0.00016
C3	-0.83235	6.70E-05	0.000165
PIK3R2	-0.12006	6.75E-05	0.000166
SPRY4	0.243104	6.92E-05	0.00017
GZMK	0.214865	7.40E-05	0.000181
TWF1	-0.12207	7.41E-05	0.000181
GPX4	-0.10541	7.52E-05	0.000183
KDM6A	-0.15781	7.67E-05	0.000187
SHC1	-0.28991	8.48E-05	0.000206
KRT10	-0.12007	8.51E-05	0.000206
ID4	-0.25324	8.60E-05	0.000208
PSEN1	-0.13012	8.82E-05	0.000213
HHEX	0.212081	9.14E-05	0.00022
LY96	0.15711	9.18E-05	0.00022

SHC2	-0.28786	9.27E-05	0.000222
RIMKLB	-0.17313	1.07E-04	0.000256
PYCR2	-0.1593	1.12E-04	0.000267
RANBP2	-0.11221	1.13E-04	0.000268
ELOB	-0.11577	1.14E-04	0.000271
SERPINA1	0.908558	1.28E-04	0.0003
HLA-E	0.252519	1.28E-04	0.0003
PLK1	-0.14871	1.36E-04	0.000318
PDGFRA	0.27936	1.55E-04	0.000361
TPSAB1/B2	0.265569	1.56E-04	0.000363
SNAI2	0.214216	1.59E-04	0.00037
CXCL10	0.282236	1.62E-04	0.000376
SLC2A1	-0.46292	1.67E-04	0.000386
RPS27A	-0.20312	1.72E-04	0.000397
SMAP1	-0.11389	1.76E-04	0.000406
H3-5	-0.1828	1.83E-04	0.00042
TCF3	-0.1306	1.84E-04	0.00042
CD40	0.175711	1.86E-04	0.000424
CEBPB	-0.13001	1.86E-04	0.000424
TDO2	0.324547	1.86E-04	0.000424
PKM	-0.19334	1.97E-04	0.000446
FAP	0.378625	2.02E-04	0.000455
CARD11	-0.11453	2.04E-04	0.000459
L1CAM	-0.27334	2.06E-04	0.000462
ZEB1	0.119228	2.14E-04	0.00048
HRAS	-0.11531	2.16E-04	0.000482
PNMA1	-0.13133	2.29E-04	0.000511
ARID2	-0.11092	2.46E-04	0.000547
BCL2L1	-0.11889	2.48E-04	0.000549
COMP	0.314497	2.62E-04	0.000579
PBX1	-0.254	2.63E-04	0.000582
BIRC5	-0.16724	2.67E-04	0.000588
SELL	0.171086	2.67E-04	0.000588
MCM2	-0.12852	2.77E-04	0.000607
SLC25A1	-0.16027	2.97E-04	0.00065
PFKFB3	0.199671	3.00E-04	0.000656
CD58	0.250883	3.19E-04	0.000695
CDC20	-0.16801	3.26E-04	0.000709
TBL1XR1	-0.28654	3.40E-04	0.000736
RUNX1T1	0.172785	3.41E-04	0.000737
KMT2C	-0.13206	3.50E-04	0.000755
NDUFB1	-0.12332	3.56E-04	0.000767
CDKN1C	-0.13547	3.62E-04	0.000778
KRT17	-0.32127	3.65E-04	0.000783

PRC1	-0.1268	3.68E-04	0.000789
DKK1	-0.23906	3.86E-04	0.000824
FEN1	-0.10968	4.04E-04	0.00086
NFKBIZ	-0.21488	4.04E-04	0.00086
HAMP	0.167328	4.08E-04	0.000867
SOCS1	0.162203	4.13E-04	0.000876
CXCL13	0.241536	4.40E-04	0.000931
PPP2R3A	-0.13228	4.46E-04	0.000942
STAT6	-0.20663	4.59E-04	0.000967
PDGFA	-0.14992	4.63E-04	0.000973
NBN	-0.10342	4.70E-04	0.000986
ATR	-0.12379	4.77E-04	0.000999
MMP9	0.610445	4.83E-04	0.00101
ETV1	0.232747	6.00E-04	0.001248
CFI	-0.33685	6.07E-04	0.00126
BRD2	-0.16593	6.16E-04	0.001278
NDC1	-0.11083	6.39E-04	0.001322
TNFAIP6	0.191442	6.49E-04	0.00134
TGFB1	0.30779	6.61E-04	0.001359
SPRY2	-0.10675	6.75E-04	0.001385
EWSR1	-0.16085	7.33E-04	0.001499
ATF2	-0.12201	8.23E-04	0.001679
CD3D	0.205263	8.74E-04	0.001774
RGMB	0.136516	9.10E-04	0.001839
DAXX	-0.10038	9.64E-04	0.001937
CDKN1A	-0.23511	9.71E-04	0.001948
ACVR2A	-0.11204	0.001037	0.002072
XCL1/2	0.138288	0.001139	0.002263
CXCL9	0.303233	0.001152	0.002284
SP1	-0.10355	0.001161	0.002297
UBE2C	-0.19705	0.00117	0.002312
PSEN2	-0.11302	0.001209	0.002385
ATRX	-0.11458	0.001245	0.002449
RPTOR	-0.11974	0.001266	0.002483
RFC4	-0.11319	0.001272	0.00249
PIM1	-0.1942	0.001287	0.002513
CD69	0.186422	0.001293	0.00252
ST6GAL1	-0.14349	0.001321	0.002571
LRP5	-0.16038	0.001351	0.002625
HLA-DOB	0.14753	0.001377	0.002666
CREBBP	-0.13323	0.001395	0.002694
SOD2	-0.48593	0.001457	0.002809
ATP5F1D	-0.12677	0.001515	0.002916
TYMS	-0.19123	0.001521	0.002923

P2RY13	0.143491	0.001531	0.002936
MMP11	0.239085	0.001536	0.002936
OAS2	0.186292	0.001537	0.002936
CD209	0.207617	0.00157	0.002983
GSK3B	-0.10419	0.001568	0.002983
MME	-0.30236	0.001566	0.002983
IL15RA	0.130705	0.001649	0.003129
RELA	-0.14096	0.001693	0.003205
CACNA2D1	0.117009	0.00183	0.003446
IRS1	0.171293	0.001848	0.003467
LTBP1	0.294029	0.001879	0.003519
RSAD2	0.151042	0.001886	0.003527
PIK3R1	0.129889	0.001926	0.003595
HLA-B	0.35059	0.002007	0.003739
SLAMF6	0.138186	0.002106	0.003918
DAB2	0.171392	0.002346	0.004355
ELK1	-0.10408	0.002648	0.004882
CD22	-0.14605	0.002708	0.004984
COL5A2	0.154931	0.002718	0.004994
IGF1	0.182797	0.002752	0.005047
RORA	0.17271	0.002764	0.005061
TSPAN7	-0.10276	0.002951	0.005393
NFATC4	0.149101	0.003007	0.005487
HES1	-0.13628	0.003016	0.005493
TNFSF10	0.234891	0.003241	0.005883
NR3C1	0.15955	0.003661	0.006598
BCAT1	-0.23775	0.003693	0.006645
MAPK12	-0.11939	0.003958	0.007109
CD44	-0.20376	0.003998	0.00717
P4HA2	-0.16101	0.004219	0.00754
SBNO2	-0.1559	0.00425	0.007582
CFD	0.161861	0.004295	0.007648
H3C8	-0.1983	0.004576	0.008122
NR4A1	0.21909	0.005001	0.008831
OAT	-0.11005	0.005144	0.009069
ARID1B	-0.13224	0.005264	0.009265
H3C2	-0.27805	0.005275	0.009269
FGF2	-0.13509	0.005288	0.009276
DTX3	-0.13446	0.005405	0.009449
TAP1	-0.18067	0.006017	0.010468
SERPING1	-0.33819	0.006251	0.010838
H3-3A	-0.19066	0.006268	0.01085
DDIT4	0.203269	0.006388	0.01104
DDB2	-0.11316	0.006463	0.011132

HLA-DPB1	0.166026	0.006656	0.011427
FOXM1	-0.12879	0.006649	0.011427
OAS1	0.153971	0.007817	0.01329
TNFSF14	-0.18377	0.007842	0.013312
DGLUCY	-0.11095	0.007898	0.013384
EGR1	0.343399	0.007934	0.013423
DEPTOR	0.150232	0.008384	0.01414
PSMB5	-0.14496	0.008501	0.014291
CCL18	0.239631	0.008761	0.014705
MYBL2	-0.17256	0.008911	0.014885
MXI1	-0.14292	0.008893	0.014885
JUNB	0.297041	0.009411	0.01567
SERINC5	-0.16093	0.010863	0.017974
MARCO	0.168362	0.010918	0.018037
FZD4	0.140201	0.01113	0.018358
MAPK14	-0.10431	0.01225	0.020078
SMO	-0.13889	0.012277	0.020091
STAT2	-0.11699	0.012471	0.020314
CCL2	0.233028	0.012528	0.020375
SPP1	0.556547	0.013807	0.022385
JAG1	0.218975	0.014322	0.023149
PCK1	-0.1968	0.014867	0.023957
RAB7A	-0.10096	0.015134	0.024349
IER3	0.169431	0.015318	0.024608
SF3B1	-0.13482	0.015632	0.025073
NLRP3	0.121291	0.015701	0.025145
CXCL2	0.173123	0.015831	0.025315
IDH1	0.112115	0.017155	0.027266
POU2AF1	0.139312	0.018255	0.028839
IL33	0.152226	0.019245	0.030266
ENO1	-0.12966	0.019349	0.030385
NOS3	0.151361	0.019689	0.030827
HELLS	-0.11028	0.019953	0.031193
CD5	0.116861	0.020487	0.031886
AMH	-0.12491	0.020794	0.032219
CLEC5A	0.174335	0.021799	0.03348
AXL	-0.19686	0.02252	0.034487
FZD1	-0.19589	0.023132	0.035372
KDR	-0.16461	0.023184	0.035386
TNFRSF25	0.106914	0.025722	0.039162
DUSP1	-0.33872	0.026359	0.040015
CXCL14	0.203815	0.026906	0.040728
ABL1	-0.17153	0.027472	0.041431
CCL15	0.223177	0.027489	0.041431

SFRP1	0.15676	0.027949	0.041824
GDF6	-0.11601	0.029467	0.043882
LGR5	0.150877	0.029988	0.044431
HSPB1	-0.12121	0.030149	0.044607
BNIP3	-0.16791	0.030259	0.044707
MYCT1	0.145242	0.030709	0.045308
IFITM2	-0.26886	0.031233	0.045823
RPS6	-0.29715	0.031573	0.046258
MKI67	-0.15588	0.03251	0.047431
IL32	0.176961	0.033709	0.048975

Supplementary Table 2: list of GO categories (S-AOIs vs E-AOIs)

	GO-BIOLOGICAL PROCESS	FRACTION OF GENES	ADJUSTED PVALUE
**GENES UP IN S vs E**	cytokine-mediated signaling pathway	83/621	4.17E-57
	cellular response to cytokine stimulus	61/482	3.03E-39
	inflammatory response	41/230	1.20E-31
	cellular response to interferon-gamma	33/121	1.20E-31
	cellular response to oxygen-containing compound	28/323	2.99E-13
	extracellular matrix organization	27/300	3.52E-13
	positive regulation of cell migration	24/269	1.18E-11
	positive regulation of cell motility	22/221	1.32E-11
	regulation of cell migration	28/408	5.74E-11
	regulation of angiogenesis	18/203	8.01E-09
	positive regulation of transcription, DNA-templated	44/1183	1.23E-08
	positive regulation of vasculature development	13/102	2.73E-08
	positive regulation of transcription by RNA polymerase II	34/908	7.88E-07
	cell-matrix adhesion	10/100	1.17E-05
	extracellular matrix disassembly	6/66	0.0014
**GENES DOWN IN S vs E**	regulation of apoptotic process	64/742	8.18E-23
	regulation of programmed cell death	46/381	7.35E-22
	regulation of cell population proliferation	57/764	1.18E-17
	mitochondrial ATP synthesis coupled electron transport	16/71	1.29E-11
	mitochondrial electron transport, NADH to ubiquinone	11/39	4.38E-09
	regulation of mitotic cell cycle	20/178	4.89E-09
	regulation of cell differentiation	23/258	1.75E-08
	regulation of mitotic cell cycle phase transition	17/188	1.80E-06
	mitotic cell cycle phase transition	17/209	6.63E-06
	regulation of cell adhesion	13/133	1.85E-05
	morphogenesis of an epithelial	8/44	2.19E-05
	apoptotic mitochondrial changes	7/33	3.40E-05
	regulation of cell adhesion	9/80	1.89E-04

cellular response to oxidative stress	11/125	2.17E-04
epidermal cell differentiation	7/53	5.17E-04
oxidative stress-induced apoptotic signaling pathway	4/18	0.0021
positive regulation of epithelial cell differentiation	4/20	0.0029

Supplementary Table 3: Full list of differentially expressed genes in pS-AOIs vs pE AOIs

Target name	***Log2 Fold Change (pS-AOIs/pE-AOIs)***	***Pvalue***	***Adjusted pvalue***
CD9	-0.7121	5.78E-07	0.000606
KRT19	-2.21512	2.94E-06	0.00108
ZEB2	0.567412	3.12E-06	0.00108
NRP1	0.560456	4.51E-06	0.00108
TGFB2	-1.71604	5.15E-06	0.00108
ABL1	-0.47597	7.16E-06	0.001131
ALCAM	-1.37571	9.11E-06	0.001131
ITGB4	-0.52457	9.70E-06	0.001131
PSMB7	-0.44332	9.67E-06	0.001131
ITGA3	-0.708	1.54E-05	0.001616
PDGFRB	0.386086	1.90E-05	0.00181
KRT17	0.105967	2.22E-05	0.001848
C4B	-2.54173	2.29E-05	0.001848
MUC1	-0.74713	3.13E-05	0.002192
IL1R1	0.133191	5.60E-05	0.003454
MS4A6A	0.824062	5.38E-05	0.003454
MIF	-0.8078	6.54E-05	0.003813
COL6A3	2.38131	8.21E-05	0.004534
SFXN1	-0.25399	9.52E-05	0.004992
RPL7A	-0.92139	0.000102	0.005084
GLS	-1.16076	0.000108	0.005156
LAMA5	-1.43205	0.000127	0.005807
GMIP	0.216553	0.00014	0.00613
HHIP	-1.2952	0.000146	0.006139
HLA-DPA1	0.89495	0.000162	0.00645
KRT18	-2.15803	0.000166	0.00645
NFE2L2	-0.27149	0.000192	0.00709
CDH3	-0.66459	0.000196	0.00709
CFB	-1.27931	0.00021	0.007207

ITGA4	0.428465	0.000229	0.007207
CD53	0.849941	0.000234	0.007207
CTSS	0.799561	0.00023	0.007207
PLOD2	-1.47426	0.000224	0.007207
ENG	0.583545	0.000271	0.008121
IL33	0.265983	0.000288	0.008395
FGF9	-0.55751	0.000304	0.008617
CIC	-0.33946	0.000325	0.00898
LCP1	0.952195	0.000343	0.009219
THY1	0.90332	0.000419	0.01098
SOCS1	0.4133	0.000431	0.011032
TNFSF10	0.676232	0.000462	0.011536
COX5B	-0.2953	0.000498	0.012146
CD4	0.785785	0.000547	0.013047
HLA-DRB	1.394209	0.000723	0.01549
CD81	-0.65648	0.00069	0.01549
NUP107	-0.30659	0.000691	0.01549
C1QB	0.879413	0.000715	0.01549
ALDOA	-0.46929	0.000724	0.01549
GPI	-0.38842	0.000744	0.015613
IL32	1.186843	0.000828	0.017031
PECAM1	0.423698	0.000863	0.017409
HLA-DRA	1.554849	0.000883	0.017482
CD63	0.308092	0.000924	0.017849
FCER1G	0.758319	0.000936	0.017849
API5	-0.2895	0.000955	0.017885
WT1	-0.41709	0.000983	0.018093
ETS1	0.544085	0.001039	0.018275
TYMP	0.746784	0.001141	0.019613
CD74	1.633992	0.001199	0.020283
C1QA	1.059668	0.00123	0.020488
JAK3	0.570569	0.001401	0.02193
C7	0.621166	0.00137	0.02193
HLA-DQA1	0.720239	0.001371	0.02193
MAP3K20	-0.80192	0.001386	0.02193
CXADR	-0.60434	0.001425	0.02198
PRDX5	-0.36382	0.001459	0.021986
FGF18	-0.50938	0.001467	0.021986
SMAD3	-0.53784	0.001489	0.021994
CXCL12	1.021535	0.001597	0.023039
ANP32B	-0.54361	0.001603	0.023039
GBP4	0.482947	0.001652	0.023421
CSF1	0.509754	0.00168	0.023493
GIMAP4	0.641213	0.001712	0.023628

LAMP1	0.329204	0.001799	0.024505
IGF1R	-0.1378	0.001884	0.025331
PCK1	-0.53454	0.001936	0.025708
CCND2	0.62909	0.002009	0.026348
ANXA1	-0.31186	0.002045	0.026489
RPL23	-0.59317	0.00211	0.026987
NLRC5	0.504206	0.002245	0.028372
GBP1	0.447898	0.002359	0.029119
MCAM	0.364252	0.002358	0.029119
SREBF1	-0.1063	0.002405	0.029337
VCAN	1.486343	0.002445	0.029477
HLA-DMB	0.787171	0.002654	0.02982
PDPN	-0.21102	0.002672	0.02982
CYBB	0.788057	0.002529	0.02982
TECR	0.342523	0.002574	0.02982
IFITM2	-0.92127	0.002615	0.02982
CARD11	-0.18141	0.002664	0.02982
CD68	1.003557	0.002605	0.02982
RPS27A	-0.36181	0.002814	0.031071
IFNGR2	0.380294	0.002901	0.0317
CD200	-0.68927	0.003026	0.032388
CD84	0.503753	0.003003	0.032388
HLA-B	1.446477	0.003146	0.032998
WIPF1	0.533989	0.003129	0.032998
PIK3CD	0.377819	0.003244	0.033689
PTPRC	0.845836	0.003324	0.034188
SPP1	-0.57925	0.003655	0.03722
BRD3	-0.46792	0.003866	0.03899
TFRC	-0.58594	0.003919	0.03907
NECTIN2	-0.63782	0.003948	0.03907
FCGR1A	0.699172	0.004247	0.041634
IFI27	-0.14019	0.004287	0.041644
MET	-0.77898	0.004511	0.043409
CD3D	0.486173	0.00459	0.043775
TRBC1/2	1.1333	0.004644	0.04389
HSPB1	0.233773	0.004782	0.044792
SLC16A1	-0.24404	0.004909	0.045571
GPSM3	0.467942	0.005086	0.046798
DNMT1	0.130718	0.005502	0.048911
ITGB2	0.679288	0.005453	0.048911
IRF8	0.49282	0.005422	0.048911
CDH2	-0.37247	0.005492	0.048911
COL27A1	-0.1501	0.005578	0.04917

Supplementary Table 4: list of GO categories (pS-AOIs vs pE-AOIs)

	GO-BIOLOGICAL PROCESS	FRACTION OF GENES	ADJUSTED PVALUE
**GENES UP IN pS vs pE**	cytokine-mediated signaling pathway	20/621	9.80E-13
	cellular response to interferon-gamma	9/121	3.59E-08
	cellular response to cytokine stimulus	14/482	3.71E-08
	T cell receptor signaling pathway	8/158	3.41E-06
	cell-matrix adhesion	4/100	0.0038
	positive regulation of angiogenesis	3/116	0.031
**GENES DOWN IN pS vs pE**	negative regulation of programmed cell death	11/381	6.90E-06
	cell-cell junction organization	6/82	3.49E-05
	cell-cell adhesion via plasma-membrane adhesion molecules	6/170	7.58E-04
	regulation of cell population proliferation	10/764	0.0021
	epithelial cell differentiation	3/101	0.024
	cellular response to oxidative stress	3/125	0.037

Supplementary Table 5: Full list of differentially expressed genes in tS-AOIs vs tE AOIs

Target name	*Log2 Fold Change (tS-AOIs/tE-AOIs)*	*Pvalue*	*Adjusted pvalue*
CSF1R	1.10914	2.42E-39	2.54E-36
FCGR3A/B	1.153437	5.45E-38	2.86E-35
A2M	1.408031	1.39E-36	3.82E-34
CTSS	0.964824	1.46E-36	3.82E-34
HLA-DMB	0.859538	2.10E-36	4.41E-34
HLA-DMA	0.857178	5.00E-36	8.75E-34
C1QA	1.465104	2.79E-34	4.18E-32
HLA-DRB	1.511025	4.42E-34	5.80E-32
C1QB	1.326793	5.32E-34	6.20E-32
WIPF1	0.553683	1.01E-33	1.05E-31
FCER1G	0.907124	1.16E-33	1.11E-31
ENG	1.036452	2.66E-33	2.32E-31
HLA-DRA	1.522005	4.27E-33	3.44E-31
HLA-DPA1	0.887067	2.49E-32	1.87E-30
ITGB2	0.773146	5.11E-32	3.57E-30
FGCR2A/B	1.174236	7.80E-32	5.11E-30
PECAM1	0.935411	1.18E-31	7.29E-30
CD14	1.222408	1.93E-31	1.12E-29

ITGA3	-1.20197	2.27E-30	1.25E-28
CD4	0.823755	7.53E-30	3.95E-28
MS4A6A	0.893596	8.10E-30	4.04E-28
CYBB	0.898197	1.19E-29	5.65E-28
HLA-DRB3	1.051696	2.31E-29	1.05E-27
LCP1	0.755256	2.03E-28	8.89E-27
CD53	0.778913	3.56E-28	1.49E-26
LAIR1	0.680223	4.30E-28	1.73E-26
CD74	1.724037	4.85E-28	1.88E-26
HLA-DQA1	0.788391	2.67E-27	9.98E-26
ZEB2	0.643603	1.31E-26	4.74E-25
LAMA5	-1.06907	6.04E-25	2.11E-23
PTPRC	0.762855	1.04E-24	3.50E-23
CD163	0.915364	1.78E-24	5.49E-23
LYZ	1.3319	1.72E-24	5.49E-23
GIMAP4	0.649961	1.75E-24	5.49E-23
TNFRSF1B	0.563233	7.95E-24	2.38E-22
KRT18	-1.55351	9.15E-24	2.67E-22
LILRB4	0.639777	1.09E-23	3.10E-22
IL10RA	0.641259	2.44E-23	6.72E-22
FCGR1A	0.689691	4.37E-23	1.18E-21
CXCL16	0.455362	1.12E-22	2.95E-21
TBXAS1	0.559124	3.54E-22	9.05E-21
PRKDC	-0.35824	4.30E-22	1.07E-20
C3AR1	0.587337	2.14E-21	5.21E-20
EPHA2	-0.52364	2.64E-21	6.29E-20
HLA-DRB4	0.79993	4.68E-21	1.09E-19
FPR3	0.464175	6.32E-21	1.44E-19
CLEC7A	0.606788	7.16E-21	1.60E-19
SPRED1	0.415766	7.90E-21	1.73E-19
KRT19	-1.52624	9.27E-21	1.98E-19
MS4A4A	0.606814	2.00E-20	4.19E-19
ITGB4	-0.93642	2.04E-20	4.19E-19
CD37	0.515784	2.82E-20	5.68E-19
LAMC2	-0.86581	3.94E-20	7.81E-19
HLA-DOA	0.552108	4.56E-20	8.86E-19
LGALS9	0.607422	1.89E-19	3.61E-18
CD68	0.751857	6.00E-19	1.12E-17
CD84	0.447495	7.25E-19	1.33E-17
TLR1	0.436588	1.35E-18	2.44E-17
CDH2	-0.68879	1.94E-18	3.46E-17
CD9	-0.65144	3.12E-18	5.46E-17
PPL	-0.60038	3.68E-18	6.33E-17
NCF4	0.498891	6.93E-18	1.17E-16

INPP5D	0.491371	7.06E-18	1.17E-16
ITGA1	0.694361	1.40E-17	2.26E-16
MRC1	0.73359	1.40E-17	2.26E-16
CD34	0.537159	1.70E-17	2.70E-16
MET	-0.67194	5.16E-17	8.08E-16
FSTL3	-0.66675	5.65E-17	8.71E-16
DSP	-0.62615	6.87E-17	1.04E-15
CFB	-1.2262	7.67E-17	1.13E-15
IGF1R	-0.50756	7.59E-17	1.13E-15
FPR1	0.426982	9.02E-17	1.31E-15
HAVCR2	0.411101	2.18E-16	3.13E-15
HCK	0.433541	4.00E-16	5.68E-15
PDGFRB	0.89574	4.74E-16	6.63E-15
TLR2	0.463847	1.03E-15	1.42E-14
GIMAP6	0.438975	1.57E-15	2.14E-14
GMIP	0.301947	1.64E-15	2.21E-14
SAMSN1	0.469576	2.07E-15	2.74E-14
THY1	0.63317	4.37E-15	5.73E-14
CSF3R	0.413956	7.10E-15	9.19E-14
CXCL12	0.535564	8.65E-15	1.11E-13
FLNC	-0.65732	9.18E-15	1.16E-13
SMARCA4	-0.31895	9.60E-15	1.20E-13
TNFSF12	0.346444	1.21E-14	1.49E-13
SLC39A6	-0.2435	1.27E-14	1.54E-13
F13A1	0.875524	1.31E-14	1.58E-13
SFRP2	0.972489	1.34E-14	1.60E-13
COL6A3	1.245364	1.46E-14	1.71E-13
NID2	0.586004	1.47E-14	1.71E-13
HSP90B1	-0.57464	2.18E-14	2.51E-13
ITGAX	0.443284	2.24E-14	2.55E-13
FZD6	-0.31945	2.39E-14	2.69E-13
JAK3	0.432649	2.44E-14	2.72E-13
RASA4	0.360224	2.47E-14	2.73E-13
COX6A1	-0.36482	2.75E-14	3.01E-13
TP53	-0.31337	3.07E-14	3.32E-13
CXADR	-0.3978	5.34E-14	5.71E-13
IRF8	0.477844	8.85E-14	9.38E-13
SIGLEC1	0.493679	9.21E-14	9.66E-13
MSR1	0.451595	1.12E-13	1.16E-12
SYK	0.391625	1.39E-13	1.43E-12
PTGER4	0.360206	1.41E-13	1.43E-12
FCGRT	0.352675	1.91E-13	1.92E-12
ITGA4	0.388346	2.39E-13	2.39E-12
SFRP4	0.693202	2.63E-13	2.61E-12

ACTA2	0.788018	3.15E-13	3.09E-12
HLA-DQB1	0.4149	4.66E-13	4.53E-12
FGF9	-0.46617	6.18E-13	5.95E-12
LILRB1	0.37341	6.83E-13	6.51E-12
TREM2	0.399659	7.06E-13	6.67E-12
GPSM3	0.327271	7.46E-13	6.99E-12
GLS	-0.53429	9.04E-13	8.39E-12
MCAM	0.584475	9.29E-13	8.55E-12
CAPN2	-0.41126	1.14E-12	1.03E-11
RAC2	0.406327	1.14E-12	1.03E-11
LY6E	-0.52372	1.42E-12	1.27E-11
C5AR1	0.381977	1.57E-12	1.40E-11
S100A9	0.723128	1.83E-12	1.61E-11
NOTCH2	-0.34221	2.27E-12	1.99E-11
MERTK	0.358646	2.36E-12	2.05E-11
ELMO1	0.361084	3.26E-12	2.80E-11
CDH5	0.455242	3.50E-12	2.99E-11
ITGAM	0.425842	4.61E-12	3.85E-11
ILF3	-0.33293	4.63E-12	3.85E-11
TLR4	0.376632	4.60E-12	3.85E-11
JAML	0.481838	6.22E-12	5.14E-11
PSMB10	0.273381	8.05E-12	6.60E-11
CD36	0.458652	1.42E-11	1.15E-10
C7	0.948297	1.83E-11	1.47E-10
ITGAL	0.398233	2.10E-11	1.68E-10
ATP2A2	-0.30873	2.22E-11	1.77E-10
LYN	0.279508	2.46E-11	1.94E-10
OLFML2B	0.545158	2.58E-11	2.02E-10
NRP1	0.600464	3.02E-11	2.35E-10
IFNGR2	0.303908	3.12E-11	2.40E-10
PRDM1	0.365617	3.59E-11	2.75E-10
LILRB2	0.548701	4.01E-11	3.05E-10
ERBB2	-0.29089	4.37E-11	3.30E-10
SLC16A1	-0.52694	5.11E-11	3.83E-10
C1S	-0.67934	5.61E-11	4.17E-10
RB1	0.193921	5.79E-11	4.28E-10
EFNA1	-0.37609	8.61E-11	6.31E-10
CCL3/L1	0.464743	8.90E-11	6.48E-10
MUC1	-0.46739	9.62E-11	6.96E-10
SKP2	-0.29517	1.31E-10	9.40E-10
CMKLR1	0.372886	1.35E-10	9.65E-10
TRAC	0.416815	1.60E-10	1.13E-09
EGFR	-0.53759	1.83E-10	1.29E-09
C4B	-1.06417	1.92E-10	1.34E-09

EFNA5	-0.335	1.99E-10	1.38E-09
PSMB7	-0.24736	4.31E-10	2.97E-09
CASP1	0.265283	4.87E-10	3.34E-09
RAD21	-0.31638	5.13E-10	3.50E-09
GAS1	-0.65513	5.79E-10	3.92E-09
NDUFA13	-0.28905	6.25E-10	4.21E-09
ITGB8	-0.2806	6.94E-10	4.64E-09
SLC11A1	0.427407	7.85E-10	5.21E-09
PRDX5	-0.25925	9.99E-10	6.59E-09
IL4R	0.251814	1.08E-09	7.11E-09
PLOD2	-0.70081	1.12E-09	7.28E-09
COL3A1	0.701836	1.14E-09	7.39E-09
RIPK3	0.266629	2.36E-09	1.52E-08
FLT1	0.396788	2.53E-09	1.62E-08
WT1	-0.44074	3.42E-09	2.18E-08
TFRC	-0.28427	4.96E-09	3.06E-08
YTHDF2	-0.16356	4.89E-09	3.06E-08
CTSH	0.330647	4.96E-09	3.06E-08
UBA7	0.187126	4.95E-09	3.06E-08
FLNB	-0.38822	4.90E-09	3.06E-08
CKLF	0.262008	5.33E-09	3.27E-08
TYMP	0.332809	5.73E-09	3.49E-08
EIF4EBP1	-0.27219	5.91E-09	3.59E-08
CDH3	-0.31618	7.12E-09	4.27E-08
IL2RB	0.352129	7.10E-09	4.27E-08
NECTIN2	-0.38641	7.18E-09	4.28E-08
HLA-DRB5	0.363444	7.36E-09	4.36E-08
IL2RA	0.381626	7.67E-09	4.52E-08
HSPA6	0.565313	9.66E-09	5.66E-08
TUBB	-0.35813	1.13E-08	6.56E-08
TLR7	0.288431	1.22E-08	7.04E-08
NDUFS8	-0.19761	1.23E-08	7.12E-08
PIK3CD	0.230218	1.26E-08	7.22E-08
VCAN	0.697927	1.28E-08	7.31E-08
APLNR	0.36643	1.37E-08	7.80E-08
CLU	-0.78652	1.46E-08	8.25E-08
REL	0.251698	1.62E-08	9.11E-08
CX3CR1	0.388757	1.77E-08	9.86E-08
C1R	-0.53685	1.81E-08	1.00E-07
TNFRSF1A	-0.22162	2.18E-08	1.21E-07
ANXA1	-0.31659	2.26E-08	1.24E-07
C2	0.321669	2.38E-08	1.30E-07
TPR	-0.18579	2.41E-08	1.31E-07
ETS1	0.282293	2.64E-08	1.43E-07

MIF	-0.46874	3.22E-08	1.73E-07
WEE1	-0.2488	3.29E-08	1.76E-07
CDK6	-0.40995	3.33E-08	1.77E-07
CSF2RB	0.296112	3.54E-08	1.88E-07
PUM1	-0.16164	3.65E-08	1.92E-07
VCAM1	0.364302	3.75E-08	1.97E-07
APP	-0.47894	3.96E-08	2.07E-07
IL1R1	0.30458	4.05E-08	2.11E-07
IL17RA	0.25974	4.09E-08	2.11E-07
COL1A2	0.696975	4.26E-08	2.19E-07
PPP2R1A	-0.2099	4.70E-08	2.40E-07
KRT5	-0.5501	4.70E-08	2.40E-07
CD46	-0.19077	4.86E-08	2.46E-07
LIF	-0.32591	5.21E-08	2.63E-07
SOD1	-0.25243	6.05E-08	3.03E-07
PRKCD	0.229021	6.69E-08	3.34E-07
TRBC1/2	0.511916	6.99E-08	3.48E-07
GTF3C1	-0.19009	7.13E-08	3.53E-07
PVR	-0.16102	8.92E-08	4.39E-07
IL2RG	0.310916	9.44E-08	4.63E-07
TNFRSF10B	-0.18898	1.02E-07	4.98E-07
S100A8	0.454512	1.04E-07	5.04E-07
ARNT2	-0.1669	1.09E-07	5.25E-07
SERINC3	-0.16412	1.13E-07	5.43E-07
TDO2	0.392132	1.23E-07	5.90E-07
DSC3	-0.282	1.34E-07	6.39E-07
BMP4	-0.39217	1.51E-07	7.19E-07
ANGPT2	0.31458	1.79E-07	8.46E-07
TMEM140	0.248305	1.80E-07	8.46E-07
ZEB1	0.200254	1.89E-07	8.84E-07
ALCAM	-0.39151	2.09E-07	9.73E-07
NUMBL	-0.16337	2.21E-07	1.03E-06
API5	-0.12228	2.22E-07	1.03E-06
CD86	0.30355	2.44E-07	1.12E-06
STING1	0.180534	2.57E-07	1.18E-06
IDH2	0.189383	2.61E-07	1.19E-06
NKG7	0.297218	2.64E-07	1.20E-06
RUNX3	0.272315	2.69E-07	1.22E-06
CCL4	0.384282	2.71E-07	1.22E-06
IL21R	0.274438	2.85E-07	1.28E-06
CXCR4	0.48438	2.86E-07	1.28E-06
B2M	0.500592	3.26E-07	1.45E-06
MCM7	-0.21009	3.82E-07	1.69E-06
NDUFB10	-0.1336	4.10E-07	1.80E-06

MST1R	-0.26646	4.11E-07	1.80E-06
MCM4	-0.18048	4.37E-07	1.91E-06
PHGDH	-0.22985	4.49E-07	1.96E-06
NDUFA7	-0.15002	4.66E-07	2.02E-06
MAP3K1	0.210306	4.77E-07	2.06E-06
FGFR1	-0.34085	5.20E-07	2.22E-06
GRB2	0.153812	5.18E-07	2.22E-06
U2AF1	-0.18533	5.17E-07	2.22E-06
CCND2	0.235942	5.47E-07	2.32E-06
TNFRSF12A	-0.3182	5.80E-07	2.45E-06
MAPKAPK2	0.119412	6.81E-07	2.87E-06
FOXC1	-0.15093	7.64E-07	3.21E-06
TGFB2	-0.53426	8.41E-07	3.51E-06
CACNB3	-0.19906	1.07E-06	4.47E-06
CCL5	0.374775	1.12E-06	4.65E-06
COL1A1	0.522294	1.16E-06	4.79E-06
CD83	0.266168	1.27E-06	5.22E-06
TOP2A	-0.28209	1.27E-06	5.22E-06
FLNA	-0.33762	1.35E-06	5.52E-06
CD200	-0.29852	1.59E-06	6.48E-06
CD48	0.279232	1.69E-06	6.85E-06
C1QBP	-0.15096	1.71E-06	6.92E-06
ASNS	-0.17428	1.75E-06	7.04E-06
BCL2	0.237863	1.83E-06	7.33E-06
LY96	0.209723	1.87E-06	7.45E-06
STMN1	-0.17921	1.92E-06	7.63E-06
PFKFB3	0.291719	2.03E-06	8.02E-06
ANLN	-0.21701	2.05E-06	8.07E-06
CD2	0.308778	2.06E-06	8.09E-06
SOS2	0.178564	2.21E-06	8.65E-06
NDUFA11	-0.1866	2.50E-06	9.75E-06
MFNG	0.221843	2.51E-06	9.76E-06
NOTCH3	0.608585	2.67E-06	1.03E-05
JAM3	0.186697	2.92E-06	1.12E-05
PDGFC	-0.21076	3.08E-06	1.18E-05
COX6B1	-0.19385	3.16E-06	1.21E-05
CCNB1	-0.17625	3.33E-06	1.27E-05
TNFRSF14	0.202535	3.34E-06	1.27E-05
JAK2	0.213609	3.38E-06	1.28E-05
NLRC5	0.199798	3.75E-06	1.41E-05
STAT3	-0.20977	3.91E-06	1.47E-05
KRT7	-0.60898	4.43E-06	1.66E-05
DIPK2B	0.285108	4.46E-06	1.67E-05
CTSW	0.231756	4.58E-06	1.70E-05

NDUFA1	-0.14164	4.63E-06	1.71E-05
AKT2	-0.17932	4.64E-06	1.71E-05
ICAM1	-0.42725	4.73E-06	1.74E-05
DAB2	0.242201	4.92E-06	1.80E-05
CACNA1C	0.1928	5.22E-06	1.91E-05
EGR2	0.259918	5.49E-06	2.00E-05
LEF1	0.28207	5.72E-06	2.08E-05
RPL23	-0.31226	6.38E-06	2.31E-05
SPRY4	0.291263	6.55E-06	2.36E-05
CCR5	0.259639	7.35E-06	2.63E-05
PTEN	0.146787	7.35E-06	2.63E-05
SERINC1	-0.15039	7.45E-06	2.66E-05
NFATC2	0.215583	7.88E-06	2.77E-05
PDGFB	0.25864	7.83E-06	2.77E-05
SRSF2	-0.15429	7.88E-06	2.77E-05
SMPD3	-0.38924	8.91E-06	3.13E-05
BST2	-0.21442	9.09E-06	3.18E-05
MDM2	-0.24947	9.58E-06	3.34E-05
NUP107	-0.1353	1.07E-05	3.72E-05
HACD2	-0.20391	1.12E-05	3.89E-05
LDHB	-0.2416	1.17E-05	4.02E-05
TET2	0.186442	1.17E-05	4.03E-05
MSH6	-0.14141	1.42E-05	4.87E-05
NFKB1	0.128765	1.43E-05	4.88E-05
GOT2	-0.10587	1.45E-05	4.93E-05
CBL	0.125182	1.47E-05	4.98E-05
PIK3R1	0.203527	1.59E-05	5.37E-05
HHEX	0.26263	1.59E-05	5.37E-05
ITGA6	-0.13097	1.63E-05	5.49E-05
PCNA	-0.1613	1.71E-05	5.72E-05
CD8A	0.250672	1.75E-05	5.84E-05
ITCH	-0.13407	1.79E-05	5.96E-05
CD209	0.252672	1.99E-05	6.59E-05
TPSAB1/B2	0.38302	1.99E-05	6.59E-05
RUNX1T1	0.190874	2.18E-05	7.18E-05
CD3E	0.260486	2.42E-05	7.95E-05
NPM1	-0.18002	2.50E-05	8.18E-05
CD55	-0.20828	2.63E-05	8.60E-05
HAMP	0.239318	2.76E-05	9.00E-05
OAS1	0.211716	3.14E-05	0.000102
HLA-E	0.245115	3.14E-05	0.000102
PLA2G2A	-0.46703	3.20E-05	0.000103
SNAI2	0.243039	3.29E-05	0.000106
NDUFB4	-0.13125	3.49E-05	0.000111

PPP2CB	-0.14036	3.47E-05	0.000111
WNT2B	-0.37588	3.48E-05	0.000111
NR3C1	0.188621	3.68E-05	0.000117
BRD4	-0.17265	4.13E-05	0.000131
KEAP1	-0.11171	4.28E-05	0.000135
COMP	0.374406	4.39E-05	0.000138
CD40	0.222637	4.62E-05	0.000145
MYD88	0.13885	5.30E-05	0.000165
ANKRD28	-0.16156	5.67E-05	0.000177
JAK1	0.100802	5.96E-05	0.000185
CDC25B	-0.18305	5.98E-05	0.000185
ADAM12	0.242638	6.27E-05	0.000193
RAC1	-0.18362	6.36E-05	0.000196
MMP9	0.724119	6.82E-05	0.000209
TAPBP	-0.10024	6.86E-05	0.00021
POLR2A	-0.11134	6.95E-05	0.000212
TNFAIP6	0.253332	6.98E-05	0.000212
IL18	-0.32705	7.16E-05	0.000217
PEBP1	-0.20799	7.19E-05	0.000217
IDH1	0.176883	7.59E-05	0.000228
FAP	0.343616	7.65E-05	0.000229
PDGFRA	0.28783	7.81E-05	0.000233
GBP4	0.227959	7.81E-05	0.000233
GZMK	0.235027	8.27E-05	0.000245
FUBP1	-0.12178	8.28E-05	0.000245
MAP3K5	0.165559	8.88E-05	0.000262
COL4A5	-0.23072	9.14E-05	0.000269
ETV1	0.248098	9.25E-05	0.000272
LFNG	0.259102	9.31E-05	0.000272
TGFB1	0.340819	9.30E-05	0.000272
OAS2	0.214342	9.58E-05	0.000279
RGMB	0.202039	0.00011	0.000318
COX5B	-0.14175	0.000111	0.000322
CDK4	-0.14288	0.000113	0.000328
MAP3K20	-0.12536	0.000114	0.000328
FGF18	-0.20341	0.000122	0.00035
IL1RAP	-0.15984	0.000127	0.000363
SAA1	-0.59426	0.000146	0.000415
RSAD2	0.218193	0.000163	0.000463
DEPTOR	0.217998	0.000165	0.000465
BRD2	-0.13031	0.000167	0.00047
POLR2H	-0.11692	0.000177	0.000498
MAP3K7	-0.10499	0.000197	0.000551
PRKAR1B	-0.17233	0.000199	0.000556

HDAC2	-0.13048	0.000206	0.000573
UBC	-0.2545	0.000212	0.000589
SERPINA1	0.862852	0.000234	0.000644
H2AX	-0.15175	0.00024	0.000657
MYC	-0.25349	0.000241	0.000658
NFATC3	0.113825	0.000248	0.000676
EWSR1	-0.12099	0.000251	0.000682
CACNA2D1	0.171247	0.000272	0.000736
RPL7A	-0.28209	0.000275	0.000743
CXCL10	0.265214	0.000295	0.000796
CD47	-0.18051	0.0003	0.000808
CCND1	-0.25319	0.000319	0.000856
SDHA	-0.12011	0.000332	0.000889
MAP2K2	-0.1251	0.000343	0.000915
SELL	0.205723	0.000348	0.000927
IRS1	0.213385	0.000373	0.000989
ANP32B	-0.13502	0.000373	0.000989
CD58	0.259111	0.000419	0.001106
BAD	-0.11784	0.000429	0.001128
CD69	0.2443	0.000457	0.001198
CENPF	-0.16383	0.000486	0.00127
GNA11	-0.10326	0.00049	0.001279
CLEC5A	0.238186	0.000496	0.001291
AXIN1	-0.11956	0.000499	0.001296
CFD	0.266642	0.000507	0.001312
CASP7	0.11293	0.000525	0.001356
HDAC1	0.120881	0.000528	0.00136
HLA-DOB	0.195557	0.000574	0.001472
MAPK3	0.110951	0.000607	0.001549
LTBR	-0.11969	0.000621	0.001581
TBL1XR1	-0.1734	0.00067	0.001698
XCL1/2	0.172636	0.000678	0.001713
SLAMF6	0.179089	0.000707	0.001775
NSD2	-0.10227	0.000707	0.001775
TAP1	-0.22032	0.000707	0.001775
MMP11	0.318985	0.000725	0.001814
LTBP1	0.307898	0.000737	0.00184
IL15RA	0.165277	0.000742	0.001845
IL6ST	-0.14738	0.000769	0.001901
RORA	0.192663	0.000767	0.001901
SPP1	0.792038	0.000784	0.001931
ALDOA	-0.345	0.000813	0.001998
C3	-0.67478	0.000851	0.002081
BRD3	-0.1233	0.00088	0.002141

DDIT4	0.257404	0.000936	0.002268
H3C2	-0.24714	0.000936	0.002268
PDPN	-0.28983	0.001016	0.002454
P2RY13	0.184421	0.001021	0.002454
CGAS	0.135387	0.001019	0.002454
IRAK4	0.127968	0.001022	0.002454
NOS3	0.217324	0.001051	0.002517
CXCL13	0.249667	0.001099	0.002627
IGF1	0.19683	0.001107	0.002639
MARCO	0.264531	0.001141	0.002708
JAG2	0.138787	0.001171	0.002773
HHIP	-0.27057	0.001217	0.00287
NOTCH1	0.178353	0.001217	0.00287
NFATC4	0.182321	0.001273	0.002993
POU2AF1	0.201652	0.00128	0.003004
JUP	-0.10514	0.001325	0.003096
IER3	0.196505	0.001332	0.003106
FZD4	0.256904	0.001392	0.003238
SOCS1	0.179492	0.001405	0.003251
CXCL9	0.327471	0.001404	0.003251
ERCC2	-0.1179	0.001407	0.003251
TNFRSF25	0.158444	0.001418	0.003264
THEM4	-0.10083	0.001442	0.00331
CASP10	0.135739	0.001456	0.003335
STAT5B	0.119547	0.001468	0.003356
HLA-DPB1	0.201771	0.001504	0.00343
PRKACB	0.156968	0.001545	0.003517
RUNX1	0.136124	0.001757	0.003947
C4BPA	-0.26713	0.001762	0.003949
CDH1	-0.17699	0.001858	0.004146
HLA-B	0.255971	0.002196	0.004891
NLRP3	0.172477	0.002248	0.004975
GPI	-0.34077	0.002376	0.005248
TICAM2	0.129036	0.002438	0.005373
SH2B2	0.154019	0.002545	0.005584
TNFSF10	0.234857	0.002873	0.006265
CCL18	0.299259	0.002938	0.006381
CD3D	0.213395	0.003027	0.00656
TNFRSF11B	-0.1464	0.003044	0.006585
CD81	-0.2167	0.003083	0.006654
JAG1	0.32479	0.003204	0.006873
CXCL14	0.268334	0.00345	0.007341
IRAK3	0.146043	0.003615	0.007677
H3-5	-0.12955	0.003624	0.00768

NFKBIZ	-0.21072	0.003824	0.008071
KRT17	-0.31613	0.00392	0.008256
COL5A2	0.179502	0.004197	0.008771
CD5	0.169978	0.00425	0.008863
SFN	-0.12298	0.004338	0.009029
NANOG	0.152803	0.00455	0.009415
CEBPB	-0.11553	0.004545	0.009415
RIMKLB	-0.12652	0.004977	0.010276
SLC2A1	-0.40295	0.005091	0.01045
UBE2C	-0.18387	0.005086	0.01045
NR4A1	0.27166	0.005106	0.010462
RRAD	-0.20045	0.005236	0.010685
CCL2	0.259225	0.00532	0.010773
ID4	-0.15452	0.00531	0.010773
SHC2	-0.17152	0.005301	0.010773
CACNG4	-0.12739	0.005348	0.01081
PKM	-0.16379	0.005628	0.011354
TNFSF13B	0.167351	0.005792	0.011662
CXCL2	0.209933	0.005808	0.011672
BAMBI	-0.12707	0.005853	0.011739
L1CAM	-0.21719	0.006291	0.01257
CD44	-0.26016	0.006417	0.012797
IRAK1	-0.12534	0.006554	0.013021
SFRP1	0.156669	0.006607	0.013102
JUNB	0.324537	0.006926	0.013683
SHC1	-0.1727	0.007025	0.013851
BCAT1	-0.28819	0.007172	0.014115
PURA	0.102511	0.007365	0.014413
RPS27A	-0.12045	0.007389	0.014417
RRAS2	-0.10199	0.007394	0.014417
ID2	0.14255	0.007508	0.014611
MYCT1	0.17381	0.00777	0.015095
ALDOC	0.167271	0.008409	0.016305
IRF2	0.102345	0.00877	0.016912
DDX58	0.108227	0.008968	0.017261
LGR5	0.157531	0.00907	0.017425
PYCARD	0.114103	0.010033	0.019206
PBX1	-0.15213	0.010144	0.019383
TGFB3	0.220825	0.010195	0.019446
ICAM3	0.136342	0.010226	0.019469
PIM1	-0.15069	0.01038	0.01969
STC1	0.265675	0.011964	0.022532
SLC25A1	-0.10283	0.012096	0.02274
SOD2	-0.4016	0.012594	0.023591

CDKN1A	-0.16661	0.01296	0.024148
HK1	0.101509	0.01341	0.024942
HDAC5	0.111649	0.013452	0.024976
FZD7	0.124073	0.013664	0.025325
DKK1	-0.15628	0.015131	0.027895
CYLD	0.103731	0.015321	0.028123
CCL15	0.189022	0.015377	0.02815
H3-3A	-0.12643	0.015479	0.028288
NGFR	0.156817	0.015953	0.029053
BIRC5	-0.10207	0.016434	0.029774
SETBP1	0.142547	0.016758	0.030309
SLAMF7	0.12887	0.017174	0.031007
IL34	0.165402	0.018516	0.033146
CDC20	-0.10918	0.018818	0.033514
IL12RB1	0.160397	0.019958	0.035424
H3C8	-0.14695	0.020192	0.035779
TGFBR1	0.10573	0.020379	0.03605
TPM1	-0.18294	0.020955	0.036944
IL7R	0.154988	0.021166	0.037192
TYMS	-0.11276	0.022607	0.039591
DUSP1	-0.32255	0.023017	0.040242
CFI	-0.22507	0.023608	0.041206
PDCD1LG2	0.123873	0.023718	0.041329
IL33	0.196439	0.023866	0.041517
FUT8	0.120268	0.024989	0.043328
ADH1A/B/C	0.301462	0.025147	0.04353
TNF	0.143829	0.025267	0.043666
SERPING1	-0.2771	0.026855	0.046107
SBNO2	-0.12616	0.027813	0.047517
AXL	-0.20523	0.028244	0.04788
CCND3	0.114919	0.028589	0.048292
EGR1	0.320636	0.029332	0.049388

Supplementary Table 6: list of GO categories (tS-AOIs vs tE-AOIs)

	GO-BIOLOGICAL PROCESS	FRACTION OF GENES	ADJUSTED PVALUE
**GENES UP IN tS vs tE**	Cytokine-Mediated Signaling Pathway	52/257	9.20E-43
	Inflammatory Response	47/236	2.59E-38
	Regulation Of T Cell Activation	20/62	2.02E-21
	Regulation Of Cell Migration	35/434	1.69E-15
	Positive Regulation Of DNA-templated Transcription	60/1243	3.18E-15
	Positive Regulation Of Transcription By RNA Polymerase II	44/938	6.52E-11
	Regulation Of Angiogenesis	18/205	3.92E-09
	Cell-Matrix Adhesion	12/109	1.37E-07
	Extracellular Matrix Organization	12/176	2.16E-05
**GENES DOWN IN tS vs tE**	Regulation Of Apoptotic Process	42/705	6.57E-16
	Regulation Of Programmed Cell Death	31/381	4.26E-15
	Regulation Of Cell Population Proliferation	39/766	4.93E-13
	Regulation Of Mitotic Cell Cycle Phase Transition	11/63	3.55E-06
	Regulation Of Epithelial Cell Proliferation	10/91	6.22E-06
	Mitochondrial ATP Synthesis Coupled Electron Transport	9/70	6.71E-06
	Regulation Of Mitotic Cell Cycle	10/125	6.25E-05
	Epidermal Cell Differentiation	6/54	7.47E-04
	Regulation Of Cell Adhesion Mediated By Integrin	5/33	7.57E-04
	Regulation Of Epithelial To Mesenchymal Transition	5/83	0.018

Supplementary Table 7: immunity related genes (Chord Plot)

CATEGORIES	GENES
**ADAPTIVE IMMUNITY**	CCL4, CCL5, CCR5, CD2, CD37, CD3D, CD3E, CD4, CD40, CD69, CD8A, GIMAP6, GZMK, IL2RA, IL2RB, IL2RG, IL32, IL4R, JAM3, PRDM1, PTPRC, SLAMF6, TGFB1, TNFRSF25, TRAC
**ANTIGEN PRESENTATION**	CD74, CD83, CTSS, HAMP, HLA-DMA, HLA-DMB, HLA-DOA, HLA-DOB, HLA-DPA1, HLA-DPB1, HLA-DQA1, HLA-DQB1, HLA-DRA, HLA-DRB, HLA-DRB3, HLA-DRB4, HLA-DRB5, ITGAX, TLR7, TREM2
**INFLAMMATION**	B2M, C5AR1, CCL15, CCL2, CD163, CD209, CD58, CLEC5A, CSF3R, CXCL10, CXCL12, CXCL13, CXCL14, CXCL16, CXCL2, CXCL9, FCGR1A, FCGRT, HCK, IDH1, IFNGR2, IL17RA, INPP5D, ITGB2, JAK3, LCP1, LILRB2, LYN, LYZ, MAP3K1, MS4A4A, MS4A6A, NFATC2, PIK3CD, RAC2, S100A8, S100A9, SLC11A1, SYK, TLR1, TLR2, TNFRSF1B, VCAN
**INNATE IMMUNITY**	CD14, CD48, CD53, CD68, CD84, CKLF, CLEC7A, CSF1R, CSF2RB, CX3CR1, CYBB, FCER1G, FPR1, FPR3, GMIP, HLA-E, HSPA6, IL1R1, ITGAL, JAML, LAIR1, LILRB1, LILRB4, LTBP1, LY96, MARCO, MRC1, MSR1, NCF4, NKG7, NLRP3, OAS1, OLFML2B, PRKCD, SAMSN1, SELL, SERPINA1, SIGLEC1, SOCS1, TLR4, TNFAIP6, TNFSF10, TNFSF12, UBA7, WIPF1
**OTHER**	A2M, C1QA, C1QB, C2, C3AR1, C7, CD86, F13A1, HAVCR2, LGALS9, TNFRSF14

Supplementary Table 8: genes included in the validation panel subdivided for categories

CATEGORIES	GENES
**ANGIOGENESIS**	ANGPT2, CXCL12, CXCR4, ENG, ETS1, FGF2, FLT1, NOS3, NRP1, PDGFA, PDGFB, PDGFC, PDGFRA, PDGFRB, PECAM1, SPRY2, TYMP, VCAM1
**INFLAMMATION/IMMUNITY**	ATF1, BAMBI, BMP4, CBL, CCL4, CCL5, CCR5, CD4, CD40, CD68, CD8A, CLEC5A, CSF1R, CTSS, CX3CR1, CXCL12, CXCL16, ICAM1, ID4, IFNGR2, IL10RA, IL32, IL33, IRF8, ITGAL, ITGAM, ITGAX, ITGB2, JAK2, JAK3, JAM3, LGALS3, MARCO, MAVS, MRC1, MUC1, MYD88, NDUFS7, PRDM1, S100A8, S100A9, SBNO2, SELL, SERPINA1, SMAD2
**OXIDATIVE STRESS**	ABL1, AKAP1, BNIP3, COX5B, COX6A1, COX6B1, FH, GPX4, NDUFS7, NDUFS8, PMAIP1, PRDX5, SDHA, SOD2, THEM4, UQCR11, UQCRQ
**SIGNALING**	BAMBI, BMP4, CMKLR1, EGFR, ERBB2, FGF9, FGFR1, GSK3B, HHIP, IGF1, IGF1R, IRS1, JAG1, JAG2, MERTK, MET, NOTCH1, NOTCH2, NOTCH3, NR3C1, OAT, PDGFA, PDGFB, PDGFC, PDGFRA, PDGFRB, RICTOR, RPTOR, SERPINB5, TGFB1, TGFB2, TSC1
**STRUCTURE/EMT**	CDH1, CDH2, CDH3, CDH5, DSC3, DSP, EFNA1, EFNA5, EPHA2, FAP, FLNA, FLNB, ICAM1, ITGA1, ITGA2, ITGA3, ITGA4, ITGA6, ITGAL, ITGAM, ITGAX, ITGB2, ITGB4, ITGB8, JUP, KRT10, KRT14, KRT17, KRT18, KRT19, KRT5, KRT7, L1CAM, LAMA5, LAMC2, MCAM, MMP9, PDPN, PPL, SNAI2, SPP1, STMN1, TGFB1, TGFB2, ZEB1, ZEB2
**TRANSCRIPTION FACTORS**	ARNT2, ATF1, ATF2, CEBPB, E2F3, EGR1, EGR2, ELK1, EP300, ETS1, ETV1, ETV4, FOXC1, HES1, HIF1A, ID4, JUNB, LEF1, MYC, MYD88, NFATC2, NFATC4, NFKB1, PBX1, PRDM1, RORA, RUNX3, SMAD2, SMAD3, SMAD4, SMAD5, SNAI2, SREBF1, STAT3, STAT6, TCF3, VHL, WT1, ZEB1, ZEB2

OTHERS	APC, ARID1B, ARID2, ATRX, AXIN1, BAP1, BRD2, BRD3, BRD4, BRD7, CREBBP, CTSH, CTSW, DKK1, ELMO1, ERCC2, FZD1, FZD2, FZD4, FZD6, HHEX, linc00941, LRP5, MAML2, MDM2, MDM4, MED12, MEN1, MXI1, NFE2L2, NR4A1, PBRM1, PSEN1, PUM1, SBNO2, SETD2, SFRP1, SFRP2, SIN3A, SMARCA4, TET2, TNKS, WNT2B, WNT5B, TET2, TNKS, WNT2B, WNT5B
